# The Chromatin Remodeler LET-418/Mi2 is Required Cell Non-Autonomously for the Post-Embryonic Development of *Caenorhabditis elegans*

**DOI:** 10.3390/jdb7010001

**Published:** 2018-12-24

**Authors:** Makhabbat Saudenova, Chantal Wicky

**Affiliations:** Department of Biology, University of Fribourg, 1700 Fribourg, Switzerland; machabbat@hotmail.com

**Keywords:** chromatin, epigenetics, development, *Caenorhabditis elegans*

## Abstract

Chromatin condition is crucial for the cells to respond to their environment. In *C. elegans*, post-embryonic development is accompanied by the exit of progenitor cells from quiescence in response to food. The chromatin protein LET-418/Mi2 is required for this transition in development indicating that proper chromatin structure in cells of the freshly hatched larvae is important to respond to food. However, the identity of the tissue or cells where LET-418/Mi2 is required, as well as the developmental signals that it is modulating have not been elucidated. By restoring the activity of LET-418/Mi2 in specific tissues, we demonstrate that its activity in the intestine and the hypodermis is able to promote in a cell non-autonomous manner the exit of blast cells from quiescence and further development. Furthermore, we identify the IIS (insulin/insulin-like growth factor signaling) pathway to be one of the signaling pathways that is conveying LET-418/Mi2 cell non-autonomous effect on development.

## 1. Introduction

Chromatin remodelers play important roles during metazoan development through their regulatory role in gene expression. They are usually part of large enzymatic complexes that modulate chromatin structure. The highly conserved, ATP- dependent chromatin remodeler, Mi2 is part of an abundant multi-protein complex in mammalian cells called NuRD (nucleosome remodeling and deacetylase) [1]. In mouse embryonic stem cells NuRD modulates the activity of pluripotency genes in a way that allow cells to respond to developmental cues [2,3]. During skin development and in the mouse hematopoietic cell lineages, Miβ is required for stem cell homeostasis and lineage choice [4,5]. Recent molecular studies showed that NuRD acts at gene regulatory sites by increasing nucleosome density and consequently decreasing the accessibility of transcription factors to these regions [6].

In *C. elegans* there are two Mi2 homologs, LET-418 and CHD-3. While *chd-3* mutant worms do not show any phenotype, worms lacking *let-418* activity present important developmental defects, such as sterility, larval arrest and vulval malformations [7,8,9]. LET-418 is also required to maintain normal lifespan and together with the histone demethylase SPR-5, the *C. elegans* homolog of LSD1, it was shown to maintain pluripotency of germ cells [10]. LET-418 also interacts with the nuclear RNAi pathway to silence repetitive elements, including transposons and retrotransposons, to ensure genome stability [11].

Detailed characterization of the *let-418* associated developmental defects revealed that post-embryonic development is not initiated and blast cells and germ cells do not divide [12]. In *C. elegans* post-embryonic development is not initiated in the absence of food and the freshly hatched L1 larvae go into a diapause stage until food is available [13]. This developmental arrest is regulated by the insulin signaling, which is sensitive to nutrient availability [14]. *let-418* L1 larvae look superficially similar to diapaused larvae and might be deficient in food sensing. The developmental arrest associated with *let-418* mutation is dependent on a network of chromatin regulators. Most of these chromatin associated proteins are part of large complexes that are involved in activation of transcription [12]. These findings highlight the importance of chromatin state in cells of the freshly hatched larvae to ensure a proper response to the environment. 

In this study, we developed molecular tools to modulate the activity of *let-418* in different tissues for the purpose of identifying LET-418 focus of action. We found that LET-418 functions cell non-autonomously in the intestine or in the hypodermis to control the onset of progenitor cell proliferation. However, to support continuous and coordinated development of all tissues, LET-418 activity is further required in the progenitor cells, as well as in adjacent tissues. Furthermore, we show that the cell non-autonomous function of LET-418 in triggering the exit of blast and germ cells from quiescence relies on the insulin signaling pathway.

## 2. Materials and Methods

### 2.1. C. elegans Growth Conditions and Developmental Assay

All the strains used in this study were maintained on agar plates containing standard nematode growth media (NGM) seeded with *E. coli* OP50 at 15 °C. To determine the number of M cell, V cell, or germ cell descendants, L4 animals carrying of the appropriate genotype and grown at 15 °C were shifted to the restrictive temperature of 25 °C. To synchronize F1 progenies, adults, after 15 h of incubation, were transferred to the fresh plates and allowed to lay eggs for two hours. F1 progenies were examined for bypass of L1 arrest by analysing the overall morphology 55 h after birth. F1 progenies were analyzed after 22, 30, 45 and 55 h after birth to determine the number of fluorescent cells (V cell and M cell descendants) and after 22, 30, 45, 55, 75, 100, 124 and 148 h after birth to determine the number of germ cells. For blast cell division analyzes, worms were paralyzed by 1 mM levamisole, mounted on 2% agarose pads and imaged on UV-light microscope. *scm::gfp* reporter was used to score the number of V lineage cells and *hlh-8::gfp* reporter to score the number of M lineage cells.

### 2.2. DNA Transformation

Improved Mos1 mediated single copy insertion (MosSCI) technique [15] was used to insert transgenes into a defined site in the *C. elegans* genome. MosSCI transformation was performed based on the protocol described on www.wormbuilder.org/test-page/protocol. The strains FR1382 (*ttTi4348* I; *unc-119(ed3)* III) or EG8081 (*unc-119(ed3)* III; *ttTi5605* IV) were used for injection. Injection mixes contained pCFJ601, pMA122, pGH8, pCFJ90, pCFJ104, and the respective expression clone (For a list of plasmids used in this study: Supplemental information).

### 2.3. Imaging and Microscopy

Microscopical analyses were performed by Zeiss Axioplan 2 microscope, as described by Erdelyi and co [12]. For brightfield pictures DIC filter, for fluorescence images the appropriate fluorescence filter was used. All images were acquired with a Zeiss AxioCam color camera driven by AxioVision v4.8.2 software (Carl Zeiss Microscopy, Jena, Germany). Images were adjusted for contrast, cropped, and merged using Adobe Photoshop.

### 2.4. Germ Cell and Blast cell Division Analyses

The germ cell division analysis was based on DAPI (2-(4-Amidinophenyl)-6-indolecarbamidine dihydrochloride) staining. For fixation and staining, rapid DAPI staining protocol was applied, as described by Käser-Pébernard and co. [10]. Worms were harvested and washed with three consecutive washes in M9 buffer, fixed for 10 min in ice-cold methanol (100%), washed three times in M9, stained with 2 µg/mL DAPI (Sigma-Aldrich, St-Louis, MO, USA) for 10 min at room temperature, washed three times in M9, and mounted in Vectashield mounting medium (Vector Laboratories, Maravai Lifescience, Chicago, IL, USA) (20 µL for 24 × 50 mm coverslip). The number of proliferative germline nuclei, of sperms and of oocytes was counted using a UV-light microscope (Zeiss Axioplan 2 microscope, Carl Zeiss Microscopy, Jena, Germany) and 4-digit manual counter clicker.

The level of blast cell divisions was assayed by *scm::gfp* reporter for seam cells and *hlh-8::gfp* reporter for the M cell [16,17]. Animals were mounted on 2% agar pad and anesthetized in a droplet of 1 mM levamisole, the number of GFP positive cells was determined using UV-light microscope. 

### 2.5. Statistical Analysis

Unpaired *t*-test was carried out using Microsoft Excel. Fisher’s exact test was performed online using web page http://graphpad.com/quickcalcs/contingency1/.

## 3. Results

### 3.1. let-418 Expression Can Be Controlled by Tissue-Specific Promoters

**Figure 1:**
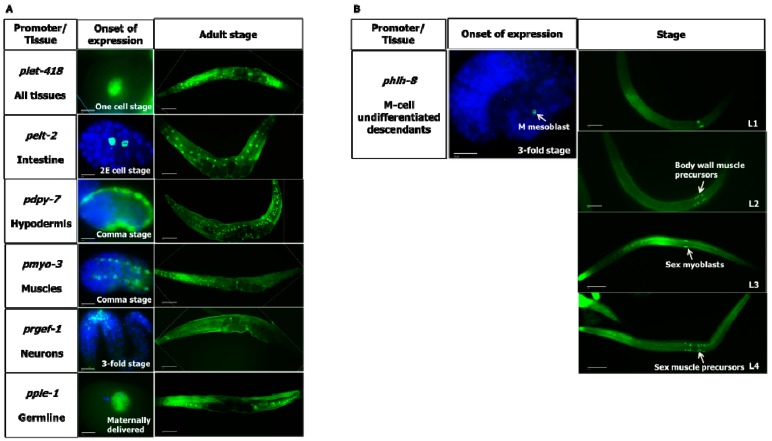
Tissue-specific expression of *let-418*. (**A**) *let-418* expression under the indicated promoter in the embryo and in the adult. (**B**) *let-418* expression under the control of the *hlh-8* promoter. DAPI in blue, antibody anti-FLAG in green. Scale bar embryo and adult 20 µm.

Loss of *let-418* maternal gene activity leads to developmental arrest at the L1 stage [9,12]. To determine in which tissues LET-418/Mi2 is required for postembryonic development, we generated transgenes including tissue specific promoters to drive expression of FLAG-tagged LET-418 in the intestine (*pelt-2*), the hypodermis (*pdpy-7*), the muscles (*pmyo-3*), the neurons (*prgef-1*), the M cell lineage (*phlh-8*) and the germline (*ppie-1*). The resulting transgenes were injected into the worm germline and integrated into the genome using the MosSCI technique [18]. Immunostaining with anti-FLAG antibody revealed that the tissue-specific promoters are driving expression of FLAG-tagged LET-418 in the appropriate tissue (Figure 1 and Supplementary Materials). Expression of *let-418* under its native promoter is observed in most if not all cells (Figure 1). Expression pattern analysis throughout development shows that *let-418* expression driven by its own promoter and by the *pie-1* promoter is seen already in one-cell stage embryos, indicating that the gene product is maternally delivered. Under the control of the *pie-1* promoter *let-418* expression persists in all cells of the embryos with declining levels after the 100 cell-stage. It is only at the larval stage that the expression gets restricted to the germ cells (data not shown). The other transgenes start to be expressed at different stages of embryogenesis (Figure 1A). *pelt-2::let-418* begins to be expressed at the 2E cell stage (endodermal cell stage) in two cells and is expressed until adulthood in intestinal cells. *pdyp-7::let-418* and *myo-3::let-418* appear at the coma stage in hypodermal cells and muscle cells respectively, and expression persists until adulthood in the corresponding tissues. *rgef-1p::let-418* is observed first at the three-fold stage of the developing embryo and in neurons of the adult worm (Figure 1A). *hlh-8p::let-418* drives expression in all undifferentiated cells of the M lineage [17] (Figure 1B). Altogether, these results show that *let-418* expression is restricted to the tissues targeted by the different promoters.

### 3.2. LET-418 Triggers Post-Embryonic Development in a Cell Non-Autonomous Manner

To identify tissues where LET-418/Mi2 is required to promote postembryonic development, we assessed the level of rescue of the different transgenes in the *let-418* mutant background. To do so, we examined the overall development of the worms and we monitored the divisions of the blast cells from the V and M lineage and of the germline precursor cells (Z2/Z3) in the different genetic backgrounds.

*let-418* expression driven by its own promoter leads to a complete rescue of the developmental defects (Figure 1A, Table 1 and [19]). This indicates an unaltered function of the LET-418 protein by the FLAG tag. *let-418* expression in the intestine or in the hypodermis is sufficient to trigger initiation of postembryonic development (Table 1). 

Based on morphological analysis, we observed that *let-418* expression in the intestine drives development until the L3/L4 stage. Animals expressing *let-418* in the hypodermis are able to bypass L1 arrest and reach the early L2 stage. *let-418* expressed under the control of the *pie-1* promoter allow the worm to develop until adulthood when the transgene is maternally provided. However, when the *pie-1p::let-418* transgene is paternally inherited, the worms arrest their development at L1. None of the other transgenes promote exit from the L1 developmental arrest (Table 1). These results show that LET-418 functions cell non-autonomously and indicate that LET-418 is required for chromatin function in the intestine and to a lesser extent in the hypodermis to initiate postembryonic development. 

To examine the level of development in different tissues, we monitored cells division from three different lineages.

#### 3.2.1. M Lineage 

**Figure 2:**
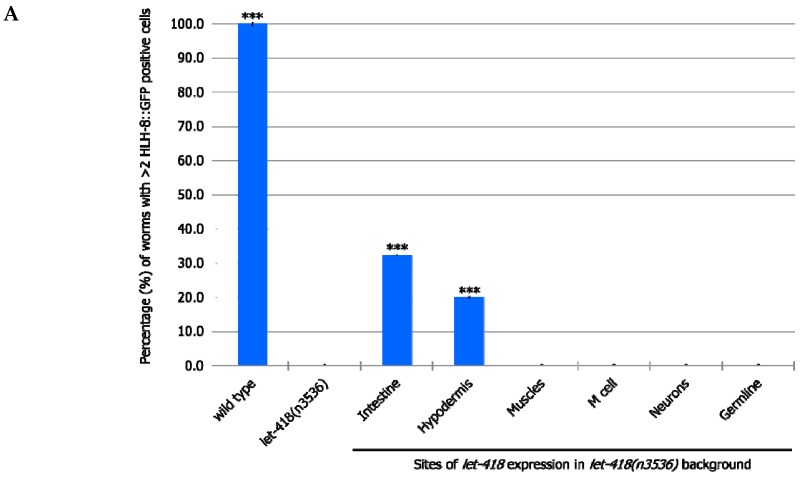

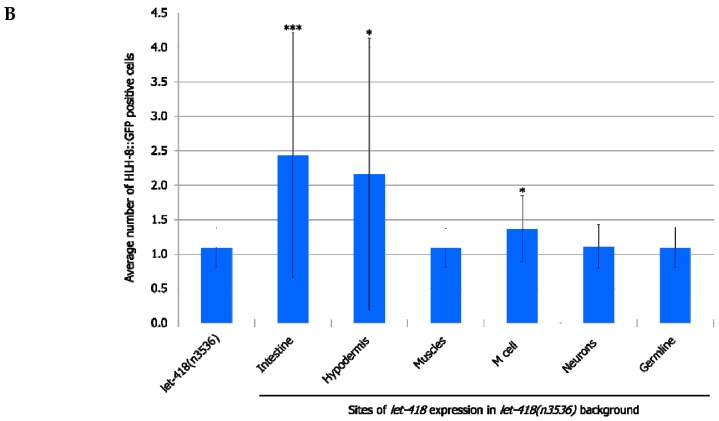
*let-418* expression in the intestine and the hypodermis triggers postembryonic development of *let-418* mutant. (**A**) The percentage of worms that showed >2 M cell divisions 30 h after birth is plotted for the different genetic backgrounds. (**B**) The average of GFP-positive cells 30 h after egg-laying is represented. * *p* < 0.05, *** *p* < 0.001; unpaired *t*-test against *let-418(n3536)*. For a better visual representation of the data the average number of M-cell descendant in wild type worm (18.7 ± 0.9) is not represented in the graph.

We examined the effect of the different transgenes on the M lineage during postembryonic development. Freshly hatched wild type L1 larvae have a single M cell, which will give birth to 2 coelomocytes, 14 body wall muscle cells and 2 sex myoblast cells [20]. The M cell lineage was visualized using a *phlh-8::GFP* reporter, which is expressed in all undifferentiated cells of the M lineage [17]. While expression of *let-418* in the neurons, muscles and germ cells has no effect on M cell division, LET-418 activity in the intestine, the hypodermis and also in the M cell is able to trigger M cell division (Figure 2, Supplemental Figure S1, Supplemental Tables S1 and S2). A small proportion of the worms show more than 2 M cell divisions at 30 h after birth when *let-418* was expressed in the intestine or in the hypodermis (Figure 2A). However, the number of GFP positive cells stays very low in these worms (Figure 2B). Animals expressing *let-418* in the M cell also show a number of M cell that is significantly higher than in *let-418* mutant (Figure 2B), however the number of animals showing more than 3 M cell descendants is not significant. This suggests that LET-418 exerts a weak cell autonomous effect on M cell division, however the M cell remains poorly responsive to developmental cues. Altogether these results indicate that LET-418 is predominantly acting cell non-autonomously in the intestine and in the hypodermis to trigger postembryonic development. 

#### 3.2.2. V Lineage

**Figure 3:**
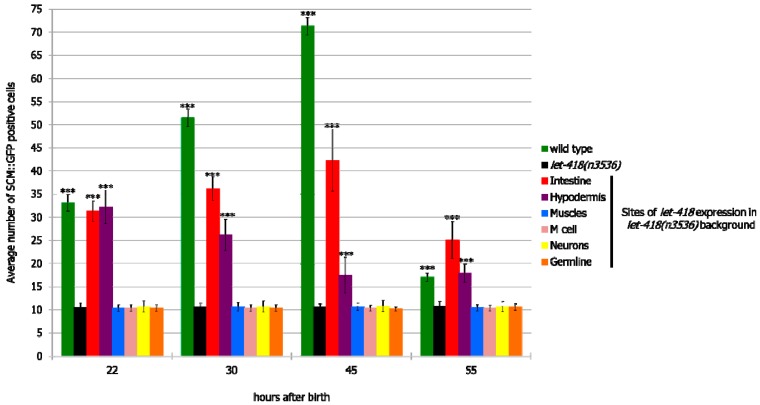
LET-418 activity in the intestine or the hypodermis triggers V cell division. Average number of V cell descendants in the indicated genetic background. Error bars indicate standard deviation. *** *p* < 0.001; unpaired *t*-test against *let-418(n3536)*.

Next, we examined the effect of LET-418 activity on cell division of the V lineage. Freshly hatched wild type larvae possess 10 pairs of seam cells belonging to the V lineage. All of them, except the most anterior ones (H0), are blast cells that will divide during development and give rise to different cell types, such as hypodermal cells, neurons and glial cells [20]. Seam cells were visualized by the seam cell specific reporter *scm::gfp* [16]. *let-418* expression in neurons, germline, M cell and muscle had no effect on V cell divisions (Figure 3 and Supplemental Figure S2). However, when *let-418* activity was restored in the hypodermis or the intestine, V cell divisions could be observed in the course of postembryonic development (Figure 3, Supplemental Figure S2 and Supplemental Tables S3 and S4). Moreover, the percentage of worms where V cells exit quiescence is higher compared to the M cell in the same genetic background (Supplemental Tables S1–S4), indicating that these cells are more responsive to the presence of LET-418 in the intestine or the hypodermis compared to cells of the M lineage. 

#### 3.2.3. Germ Cells

**Figure 4:**
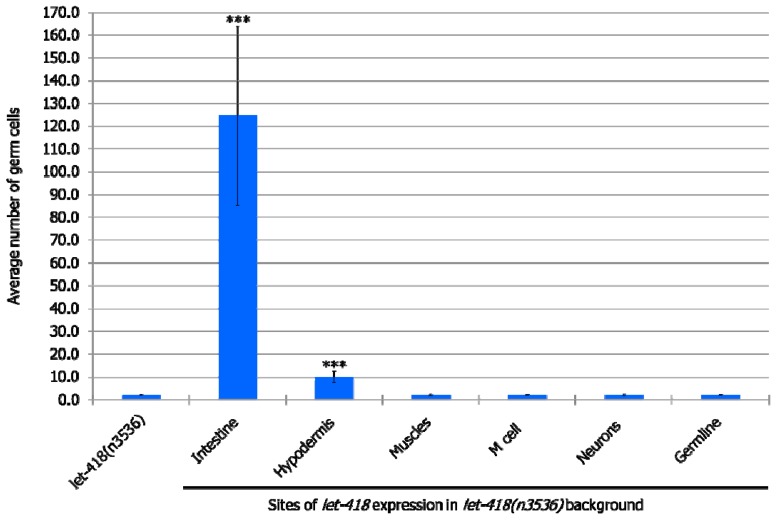
LET-418 activity is required in the intestine and the hypodermis for germ cell proliferation. The highest average number of germ cells is plotted for the indicated genetic backgrounds. For clarity purposes wild worms, which show an average of 1024 ± 24.2 germ cells at 124 h after birth is not represented on this graph. Error bars indicate standard deviation. *** *p* < 0.001; unpaired *t*-test against *let-418(n3536)*.

At the non-permissive temperature of 25 °C, in the *let-418* mutant, Z2 and Z3 germline precursor cells are quiescent. When LET-418 activity is present in the intestine, germ cell proliferation is observed to a level that corresponds to the L3 stage in wild type worms (Figure 4, Supplemental Figure S3, Supplemental Tables S5 and S6). Furthermore, a small proportion of germ cells could differentiate into sperms (Supplemental Table S6). We also noted that almost all the worms exhibit Z2/Z3 cells that exit quiescence, indicating that these cells are highly responsive to the presence of LET-418 in the intestine (Supplemental Table S5). When *let-418* activity is restored in the hypodermis, very low level of Z2/Z3 proliferation is observed (Figure 4, Supplemental Figure S3 and Table S6). No proliferation was observed when *let-418* activity was restored in neurons, muscles, M lineage and germline (zygotic expression) (Figure 4, Supplemental Figure S3, Tables S5 and S6). To get *let-418* zygotic expression in the germline, the transgene was introduced through the male germline to avoid the maternal contribution, which is sufficient to fully rescue the *let-418* mutant phenotype (data not shown). 

Taken together, this suggests that LET-418 dependent chromatin conformation in the intestine is playing a critical role in the developmental control of the entire organism, and in the signaling towards other tissues of the worm. However, lack of correspondence between M, Z and V lineage development in the different strains suggests that LET-418 has also a role in the other tissues to ensure coordinated development. 

### 3.3. Intestine and Hypodermis Are Primary LET-418 Sites of Action to Sustain Coordinated Development

**Figure 5:**
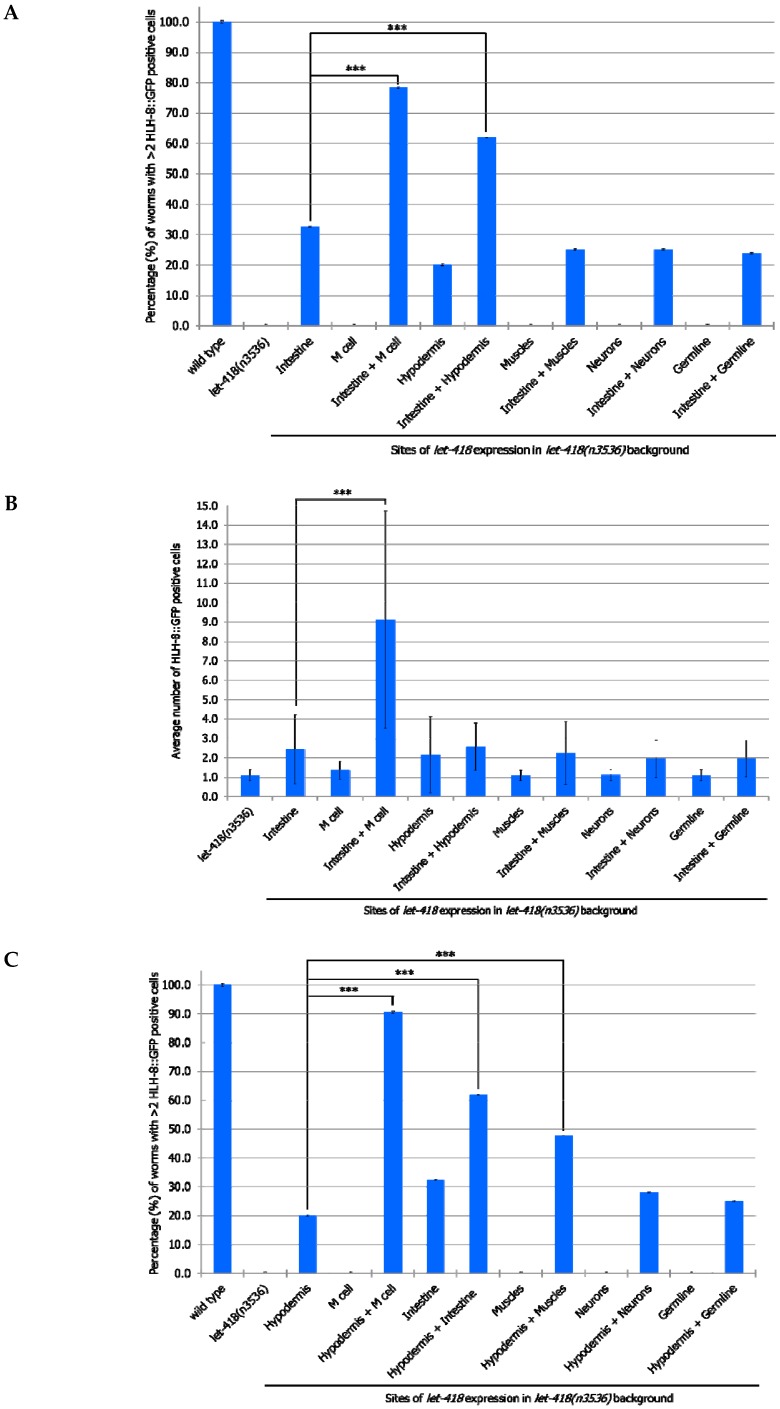

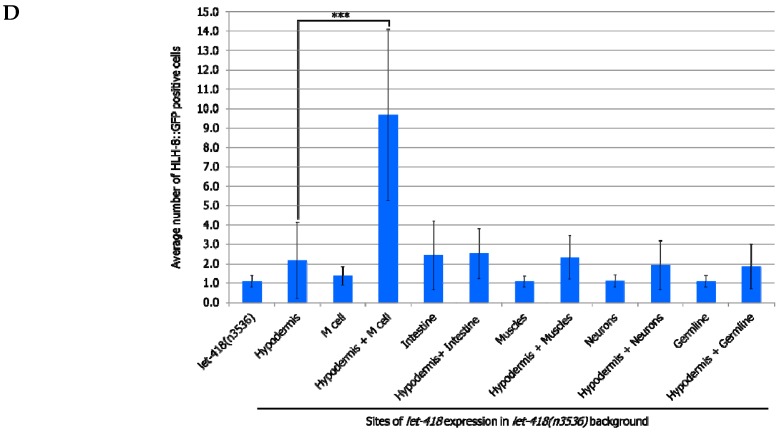
LET-418/Mi-2 acts cell autonomously in M mesoblasts for further development. (**A**,**C**) Percentage of worms with at least 3 M derived cells after 30 hrs after birth. Error bars indicate standard deviation. *** *p* < 0.001; Fisher’s test. (**B**,**D**) The average number of *phlh-8::gfp* positive cells in *let-418* mutants with LET-418/Mi-2 activity in the intestine or hypodermis combined with any of the other tissues. The average number was recorded at 30 h after birth. For clarity purposes the highest average number of M-cell descendants in wild type animals (18.7 ± 0.9) is not displayed on this graph. Error bars indicate standard deviation. *** *p* < 0.001; unpaired *t*-test.

If LET-418 is required for coordinated development, then combined expression of *let-418* from two different tissues should promote further development. In a first set of experiments, we combined all our strains expressing *let-418* from different promoters with the one expressing *let-418* from the intestine and we monitor the effect on M cell division. To do so, we generated heterozygotes worms expressing *let-418* from two different promoters (intestinal promoter and any other promoter). Expressing *let-418* in the intestine and the M cell improve the M cell ability to exit quiescence, as well as the number of divisions (Figure 5A,B, Supplemental Tables S7 and S8). This shows that LET-418 has also a cell autonomous effect on M cell development, although a primary trigger should come from the intestine. This result is consistent with the very small cell-autonomous effect which was already observed when *let-418* is expressed solely in the M cell (Figure 2B). *let-418* expression in both the intestine and the hypodermis leads to a more efficient exit from quiescence. However, there is no significant improvement in the number of M cell descendants, indicating that LET-418 activity is needed in the corresponding tissue to sustain development. No improvement in M cell exit from quiescence and division is observed in the worms expressing *let-418* in the neurons, the muscle and the germline in addition to the intestine. Next, we combined our different strains with the one expressing *let-418* in the hypodermis and assayed the effect on M cell exit of quiescence and division. Expression of *let-418* in the following combinations tissues: hypodermis/M cell, hypodermis/intestine and hypodermis/muscle could trigger M cell exit from quiescence (Figure 5C,D, Supplemental Tables S9 and S10). However, a significant level of M cell division was only observed when *let-418* was expressed in the hypodermis and the M cell. This result is consistent with the requirement of a LET-418 cell-autonomous effect in the M cell.

Altogether, we show here that the intestine and the hypodermis are primary sites for LET-418 function to promote M cell exit of quiescence. However, division of the M cell is further enhanced when *let-418* is expressed in the M cell, indicating that LET-418 also has a cell-autonomous function.

### 3.4. Germline Development is Enhanced When let-418 is Expressed in More Than One Tissue

**Figure 6:**
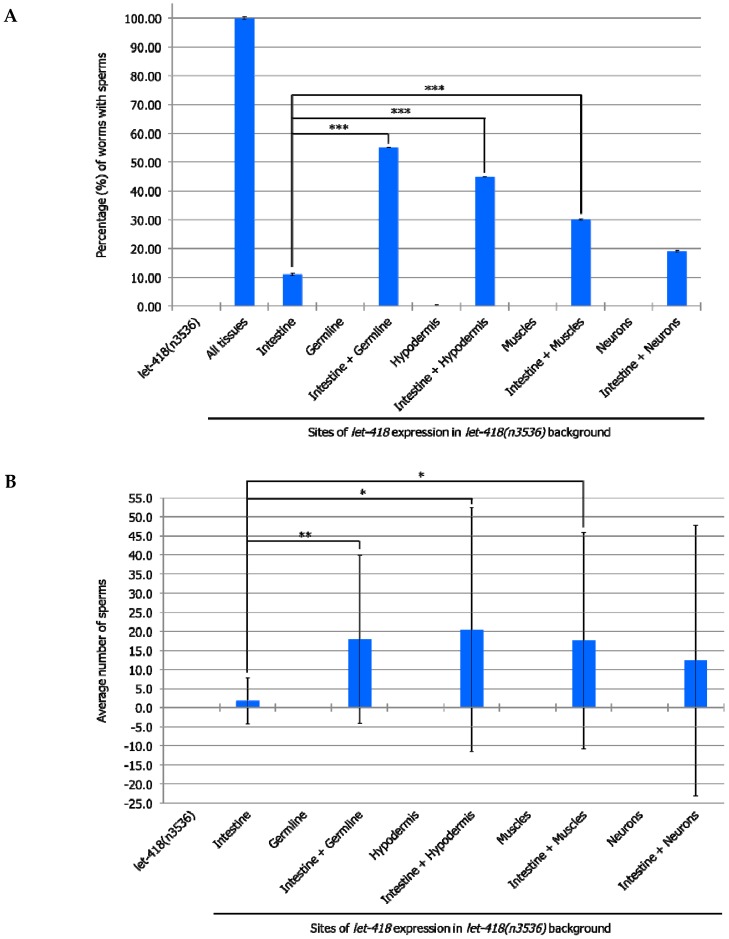
LET-418 is required in multiple tissues during germline development. (**A**) The percentage of worms with sperms after 75 h after birth. Error bars indicate standard deviation. * *p* < 0.05; ** *p* ≤ 0.01; *** *p* < 0.001; Fisher’s test. (**B**) The average number of sperms 75 h after birth. For clarity purpose we omitted the strain expressing *let-418* in all tissues. Error bars indicate standard deviation. * *p* < 0.05; ** *p* ≤ 0.01; *** *p*< 0.001; unpaired *t*-test.

To test if combined expression of LET-418 in two different tissues could also improve germline development, we generated strains where *let-418* was expressed in the intestine and in additional tissues. Enhanced level of germ cell proliferation and differentiation into sperms was observed when *let-418* is expressed in the intestine as well as in the germline (Figure 6A,B and Supplemental Tables S11 and S12). Interestingly, combined *let-418* expression in intestine and any of the other somatic tissues, except neurons, also improves germline development (Figure 6A,B and Supplemental Tables S11 and S12). Moreover, the development of the somatic gonad is also enhanced in these worms, which show further extension of gonadal arms (Supplemental Figure S4). Combined *let-418* expression in the hypodermis and any of the other tissues shows comparable results to those obtained with combinations including *let-418* intestinal expression (Supplemental Table S13). These results indicate that germline development strongly depends on LET-418 activity in all the somatic tissues, except the neurons, with a predominant effect of the intestine and the hypodermis.

### 3.5. The Cell Non-Autonomous Effect of LET-418 on M Cell Division Depends on the Insulin Signaling Pathway

We show that LET-418 activity in the intestine has the strongest effect on germ cell and blast cell proliferation, suggesting that generation of a pro-developmental signal depends on the presence of LET-418 in the intestine. Insulin signaling is known to be implicated in post-embryonic development in *C. elegans* [21,22,23]. 

To analyze whether this signaling pathway could be mediating intestinal LET-418 function on M cell division, we used the temperature-sensitive, dauer-constitutive mutant, *daf-2(e1370),* where the function of the DAF-2/insulin receptor is strongly reduced at restrictive temperature. To test the interaction between LET-418 function and the insulin signaling pathway, we generated strains where *let-418* was expressed in the different tissues in a *daf-2(e1370)* mutant background and monitored M cell division. 

**Figure 7:**
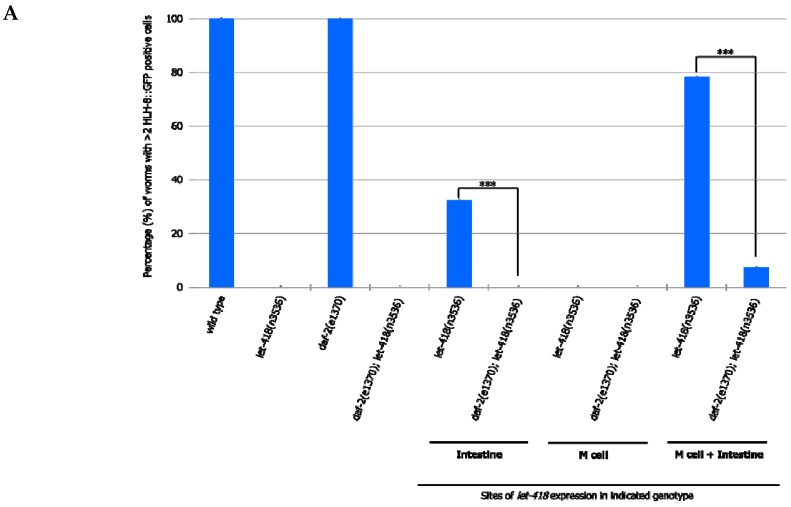

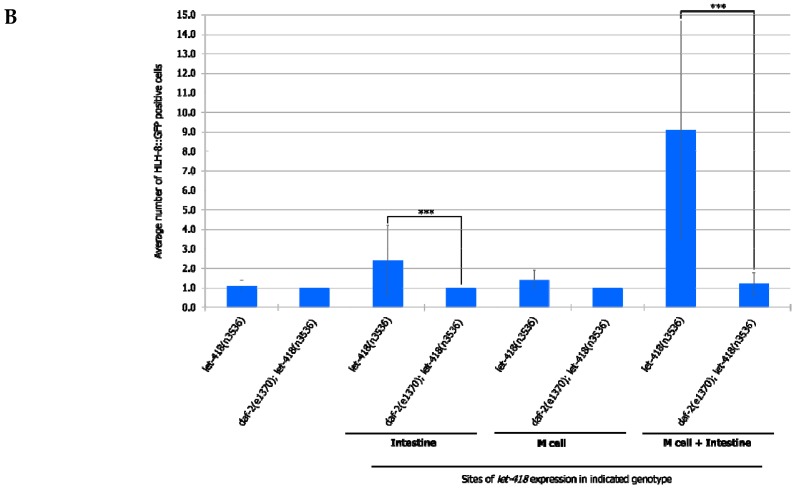
Insulin signaling is required for the cell non-autonomous effect of LET-418/Mi-2 on M cell division. (**A**) The percentage of worms with at least three M derived cells after 30 h after birth. Error bars indicate standard deviation. *** *p* < 0.001; Fisher’s test. (**B**) The average number of undifferentiated M lineage cells. Animals with intestinal LET-418 activity, including those with combined LET-418/Mi-2 activity in the intestine and M cell showed the average maximum of *phlh-8::gfp* positive cells after 30 h after birth. For clarity purpose the average number of M-cell descendants in wild type worms (18.7 ± 0.9) and in *daf-2(e1370)* mutants (17.1 ± 1.2) is not displayed on this graph. Error bars indicate standard deviation. *** *p* < 0.001; unpaired *t*-test.

In *daf-2* mutants grown at restrictive temperature, the M cell produces 14 BWMs, 2 CCs and 2 SMs, which migrate to the presumptive vulva region but fail to divide further (Supplemental Figure S5, Tables S14 and S15). This result indicates that *daf-2* activity is not required for the M cell to exit quiescence. In *daf-2;let-418* double mutant post-embryonic development does not initiate. When LET-418 activity is restored in the intestine, in reduced insulin signaling conditions, M cell division is abolished (Figure 7A,B). These worms exhibit only one *phlh-8::gfp* positive cell and are noticeably smaller in body size compared to worms with wild type DAF-2 activity (data not shown). Consistently, M cell division is also strongly reduced, however not totally abolished, in worms with combined LET-418 activity in the intestine and in M cell in a *daf-2* mutant background (Figure 7A,B). This observation is consistent with a very small cell-autonomous effect of LET-418 in the M cell (Figure 2B). Altogether, these results indicate that the cell non-autonomous activity of LET-418 relies on insulin signaling to trigger M cell division.

## 4. Discussion

Our study of LET-418/Mi2 tissue-specific contribution reveals both cell non-autonomous and cell autonomous inputs. LET-418/Mi2 plays a major role in the intestine and the hypodermis for the development of the whole organism and its impact on other tissues is mediated by the insulin signaling pathway. 

**Figure 8:**
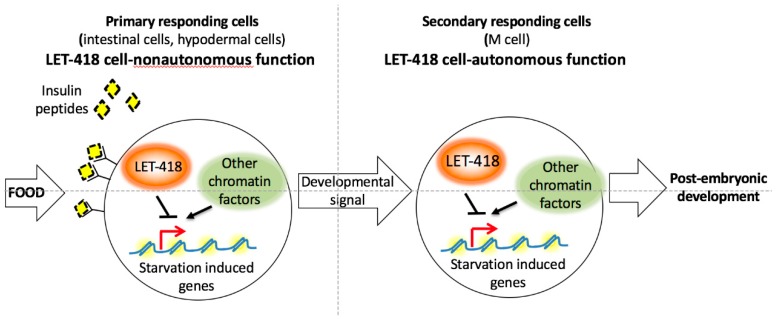
Model of LET-418 function in the transition to post-embryonic development (for more details see text).

LET-418 activity in the intestine and to a lesser extend in the hypodermis restores the ability of the worm to initiate post-embryonic development. LET-418 focus of action is in agreement with a role in the food sensing pathway. In the absence of *let-418* activity, the L1 arrested larvae look superficially similar to L1 diapaused larvae. The fed larvae might fail to integrate the resulting growth signal at the level of the intestinal chromatin (Figure 8). The intestinal chromatin might be in a state that constitutively expresses starvation induced genes leading to a developmental arrest and preventing an appropriate response to the nutrient status. 

When LET-418 function is restored in other tissues than the intestine and the hypodermis, no rescue of the developmental arrest is observed, suggesting that LET-418 is not required primarily in these tissues for development. However, we cannot rule out that the promoters used in our study do not induce sufficient expression to trigger development.

In mouse embryonic stem cells (ESCs), Mi2, as well as other NuRD components are required for a proper response to developmental cues. In the absence of Mi2, pluripotency genes are misregulated and lineage commitment of the ESCs is compromised [2,24]. LET-418 could play a similar role in *C. elegans* by shaping the chromatin of intestinal and hypodermal cells to allow them to respond to food. Accordingly, LET-418 is also required in blast cells and germ cells to allow them to respond to the signal coming from the intestine and the hypodermis.

Altogether, these results suggest that the chromatin context in the appropriate tissue is very important to respond to developmental or environmental cues. In line with this proposition, in a genome wide RNAi screen for suppressors of *let-418* developmental defects, we identified mainly chromatin regulators [12]. Absence of both LET-418 and one of these chromatin factors was able to restore initiation of postembryonic development. It would be interesting to know if these chromatin factors are shaping the chromatin at common loci with LET-418 in the different tissues (Figure 8). 

The insulin signaling pathway is required for the cell non-autonomous function of LET-418. Insulin signaling is known to be activated in response to food and to promote larval development [14]. It is thought to monitor the internal nutrient status in the intestine and to sense external nutrient status through the neurons [23,25]. We propose that in the *let-418* L1 larvae insulin signaling is initiated normally in response to food, but no appropriate transcriptional response is established in the intestine and the hypodermis, because chromatin is not in the proper state. LET-418 function in the intestine and the hypodermis would be important for the response to the nutrient status but also to "organize" the signaling to the other tissues. However, the nature of the developmental signal sent by the intestine has to be different (see below) from the insulin signaling, since we observe no response of the other tissues, when LET-418 activity is restored in these tissues. 

The DAF-16 FOXO transcription factor is known to integrate the input of the insulin signaling pathway, which antagonizes its function [26,27]. DAF-16 regulates post-embryonic development in the intestine in a cell non-autonomous manner. DAF-16 promotes L1 arrest by inhibiting *daf-12/*NHR and *dbl-1*/TGFβ Sma/Mab signaling [22]. In the absence of LET-418, the chromatin context could allow DAF-16 to activate starvation induced genes and trigger developmental arrest. Consistent with this hypothesis, we identified a significant enrichment in DAF-16 target genes that were upregulated in *let-418* L1 arrested larvae [12]. Moreover, in mouse embryonic stem cells, Mi2 was shown recently to maintain nucleosome density to control the accessibility of specific transcription factors [6]. However no bypass of L1 arrest is observed in *daf-16;let-418* double mutant suggesting that DAF-16 would contribute only partly to the *let-418* L1 arrest (data not shown).

To summarize, we showed that LET-418 functions cell non-autonomously in the intestine and the hypodermis in the transition to post-embryonic development. Its main role in the energy centers of the worm is consistent with a potential role in food sensing, which is a prerequisite for the initiation of larval development. The cell non-autonomous function of LET-418 is mediated by insulin signaling. Identifying LET-418 tissue-specific target genes will allow to characterize further the link between chromatin and the response to food.

## Figures and Tables

**Table 1:** LET-418 regulates L1 arrest cell non-autonomously.

Tissue Expressing LET-418	Promoter	Final Developmental Stage
all	*let-418*	fertile adults
intestine	*elt-2*	arrested dpy-like L3-L4 larvae
hypodermis	*dpy-7*	arrested L2 larvae
neurons	*rgef-1*	arrested L1 larvae
muscles	*myo-3*	arrested L1 larvae
M mesoblast	*hlh-8*	arrested L1 larvae
embryo, germline	*pie-1* (m+)	sterile adult
germline	*pie-1* (p+)	arrested L1 larvae
no		arrested L1 larvae

m+: maternal contribution of the transgene; p+: paternal contribution of the transgene.

## Supplementary Materials

The following are available online, Supplemental Figures and Tables (Figures S1–S5, Tables S1–S15), Supplementary materials. (Plasmid and strain lists).
