# Supplementary material for: The Chromatin Remodeler LET-418/Mi2 is Required Cell Non-Autonomously for the Post-Embryonic Development of Caenorhabditis elegans
Source: J Dev Biol. 2018 Dec 24;7(1):1. doi: 10.3390/jdb7010001 (PMC6473691; doi:10.3390/jdb7010001)
Supplement: Supplementary file 1 [file jdb-07-00001-s001.zip › jdb-399122-SI/Sup Fig and Tables.pdf]

Supplemental Figure S1

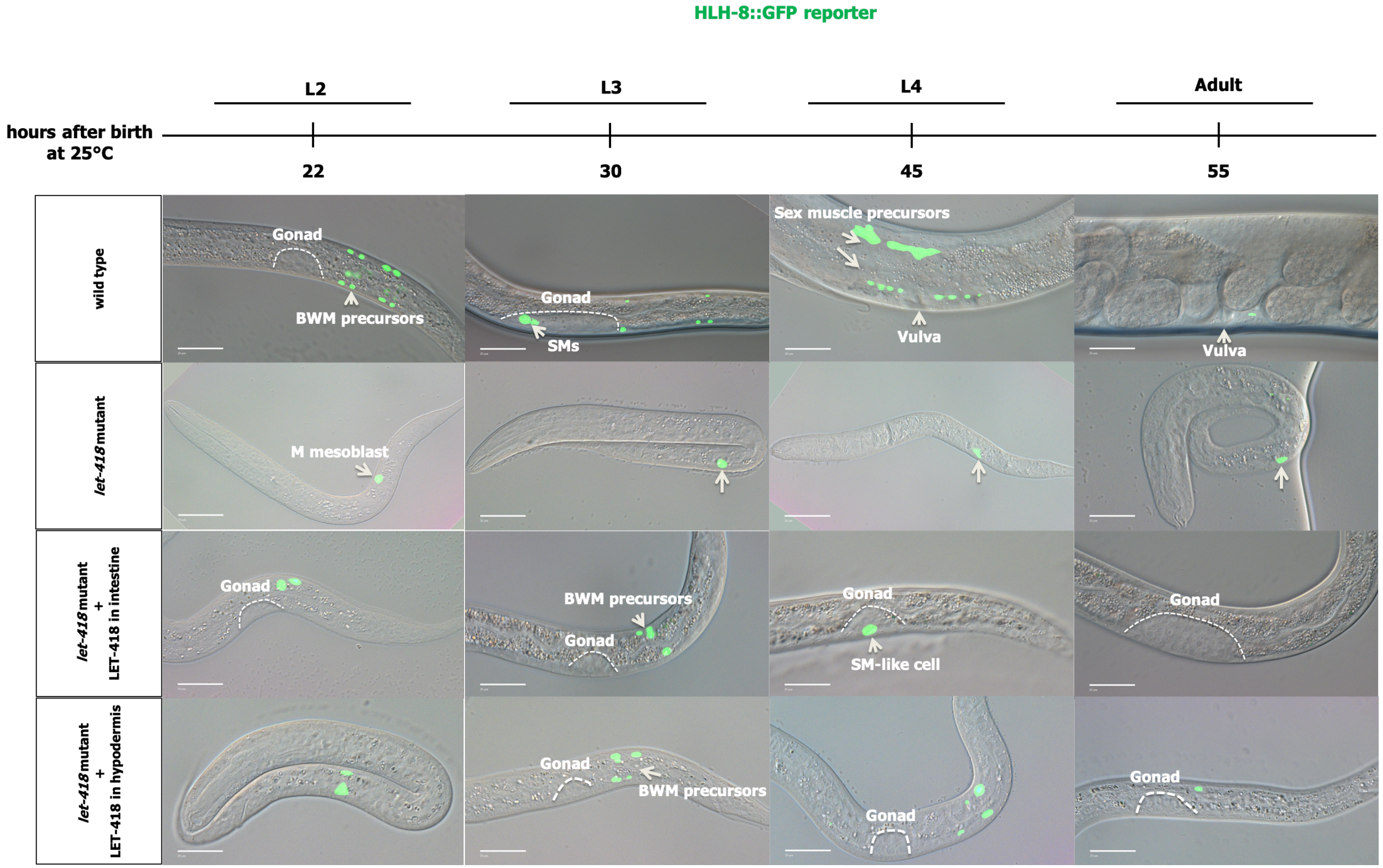

Supplemental Figure S1. LET-418 is required in the intestine or the hypodermis to trigger post-embryonic development of *let-418* mutant. Representative pictures are shown for the indicated genetic background. Undifferentiated cell descendant of the M lineage were visualized using *hlh-8::gfp* reporter in 22, 30, 45 and 55 hours after birth. During postembryonic development of wild type worms, M mesoblast undergoes a series of divisions giving rise to BWMs, CCs and sex muscles. In *let-418* L1 larvae, including those with *let-418* expression in muscles, neurons, germ cell precursors or M mesoblast, the M cell does not divide. Worms exhibiting *let-418* expression in the hypodermis or intestine rescue show low levels of M cell division, producing precursors of BWM and sex myoblast-like cells. Scale bar: 20 µm.

Supplemental Table S1

Percentage of worms exhibiting 1-2 or more HLH-8::GFP positive cells								
Strains	Hours after birth (hrs)							
	22 hrs		30 hrs		45 hrs		55 hrs	
	1 or 2 cells	>2 cells	1 or 2 cells	>2 cells	1 or 2 cells	>2 cells	1 or 2 cells	>2 cells
wild type	-	**100.0% (20)**	-	**100.0% (23)**	-	**100% (20)**	33.3% (7)	**19.0% (4)**
let-418(n3536)	100.0% (21)	-	100.0% (21)	-	100.0% (23)	-	100% (22)	-
let-418(n3536) + LET-418 in intestine	100.0% (30)	-	67.6% (25)	**32.4% (12)**	58.8% (20)	**11.8% (4)**	32.6%(14)	**4.7% (2)**
let-418(n3536) + LET-418 in hypodermis	100.0% (22)	-	80.0% (20)	**20.0% (5)**	38.8% (19)	**10.2% (5)**	21.6% (8)	**2.7% (1)**
let-418(n3536) + LET-418 in muscles	100.0% (22)	-	100.0% (35)	-	100.0%(30)	-	95.8% (23)	-
let-418(n3536) + LET-418 in neurons	100.0% (27)	-	100.0% (22)	-	100.0% (21)	-	85.7% (18)	-
let-418(n3536) + LET-418 in germline	100.0% (21)	-	100.0% (21)	-	100.0% (33)	-	90.9% (20)	-
let-418(n3536) + LET-418 in M cell	100.0% (33)	-	100.0% (30)	-	100.0% (21)	-	93.3% (28)	-

Supplemental Table S2

Average number of HLH-8::GFP positive cells				
Strains	Hours after birth (hrs); number of worms (n=)			
	22 hrs (n=)	30 hrs (n=)	45 hrs (n=)	55 hrs (n=)
wild type	**16.3 ± 1.2 (20)**	**18.0 ± 0.0 (23)**	**16.0 ± 0.0 (20)**	1.3 ± 1.4 (21)
let-418(n3536)	1.1 ± 0.3 (21)	1.1 ± 0.3 (21)	1.1 ± 0.3 (23)	1.1 ±0.3 (22)
let-418(n3536) + LET-418 in intestine	1.1 ± 0.3 (30)	**2.4 ± 1.8 (37)**	1.4 ± 1.8 (34)	0.6 ± 0.9 (43)
let-418(n3536) + LET-418 in hypodermis	1.2 ± 0.4 (22)	**2.2 ± 2.0 (25)**	0.8 ± 1.1 (49)	0.4 ± 0.8 (37)
let-418(n3536) + LET-418 in muscles	1.1 ± 0.3 (22)	1.1 ± 0.3 (35)	1.0 ± 0.2 (30)	1.0 ± 0.4 (24)
let-418(n3536) + LET-418 in neurons	1.1 ± 0.3 (27)	1.0 ± 0.2 (22)	1.0 ± 0.2 (21)	1.0 ± 0.5 (21)
let-418(n3536) + LET-418 in germline	1.1 ± 0.3 (21)	1.1 ± 0.3 (21)	1.1 ± 0.3 (33)	1.1 ±0.5 (22)
let-418(n3536) + LET-418 in M cell	1.2 ± 0.4 (33)	**1.4 ± 0.5 (30)**	1.2 ± 0.4 (21)	1.2 ± 0.5 (30)

Bold: p values <0.05

Supplemental Figure S2

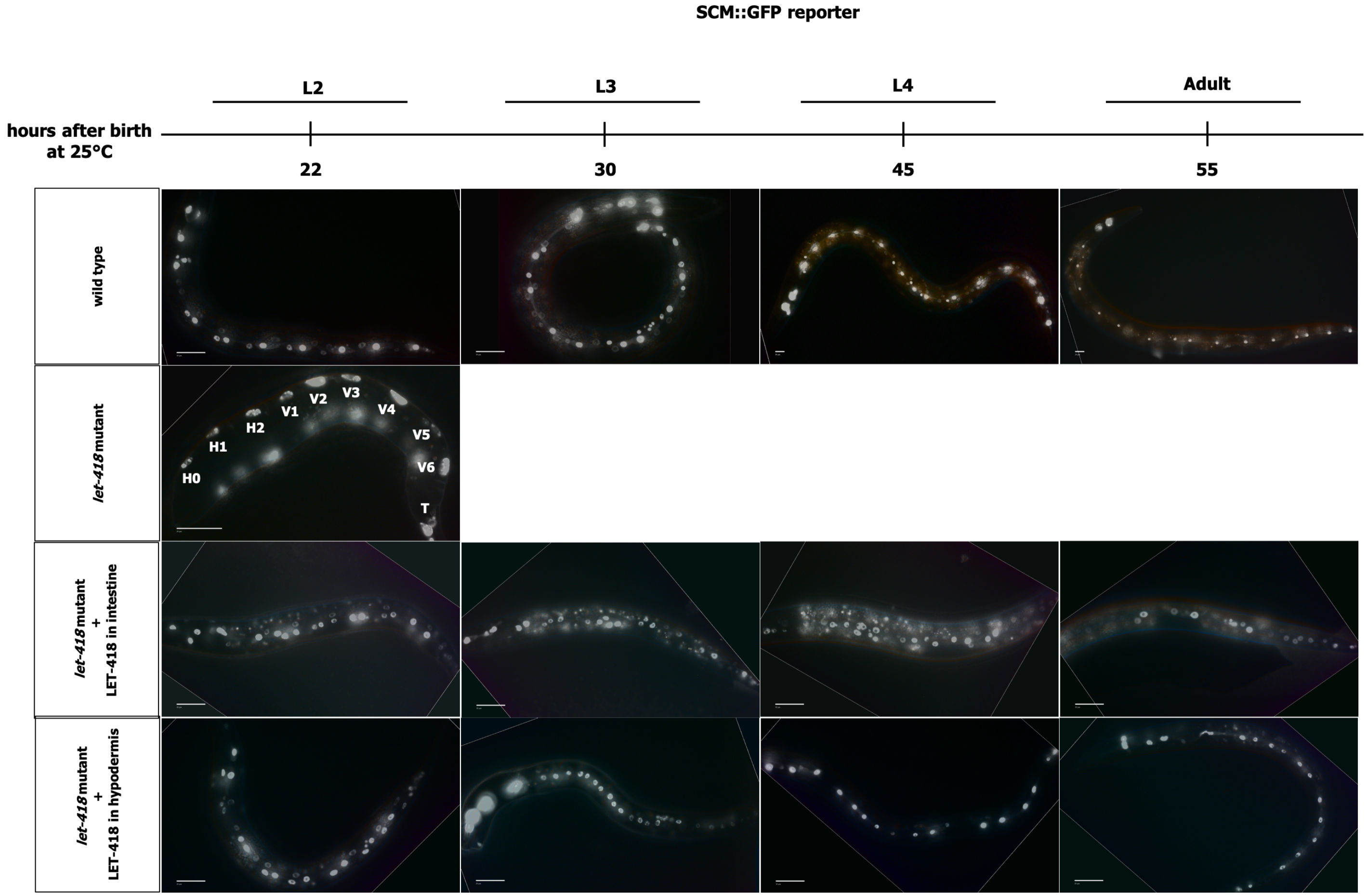

Supplemental Figure S2. Intestine-specific or hypodermis-specific *let-418* expression partially rescues seam cell divisions in *let-418(n3536)* mutants. Seam-cell lineage tracing of wild type and *let-418(n3536)* animals exhibiting *let-418* expression in the indicated tissues. Seam cells were visualized by the seam cell specific reporter *scm::gfp* 22, 30, 45 and 55 hours after birth. In *let-418(n3536)* worms, including those with *let-418* expression in neurons, muscles, M cell or germline seam cells fail to divide. Scale bar: 20 µm.

Supplemental Table S3

Percentage of worms exhibiting 10-14 or more SCM::GFP positive cells								
Strains	Hours after birth (hrs)							
	22 hrs		30 hrs		45 hrs		55 hrs	
	10 - 14 cells	>14 cells	10 - 14 cells	>14 cells	10 - 14 cells	>14 cells	10 - 14 cells	>14 cells
wild type	-	**100% (32)**	-	**100% (20)**	-	**100% (21)**	-	**100% (21)**
let-418(n3536)	100% (21)	-	100% (25)	-	100% (20)	-	100% (22)	-
let-418(n3536) + LET-418 in intestine	-	**100% (24)**	-	**100% (21)**	-	**100% (38)**	-	**100% (30)**
let-418(n3536) + LET-418 in hypodermis	-	**100% (37)**	-	**100% (20)**	3% (1)	**97% (37)**	4% (1)	**96% (26)**
let-418(n3536) + LET-418 in muscles	100% (33)	-	100% (20)	-	100% (21)	-	100% (20)	-
let-418(n3536) + LET-418 in neurons	100% (21)	-	100% (23)	-	100% (21)	-	100% (25)	-
let-418(n3536) + LET-418 in germline	100% (33)	-	100% (21)	-	100% (25)	-	100% (25)	-
let-418(n3536) + LET-418 in M cell	100% (21)	-	100% (21)	-	100% (23)	-	100% (21)	-

Supplemental Table S4

Average number of SCM::GFP positive cells				
Strains	Hours after birth (hrs); number of worms (n=)			
	22 hrs (n=)	30 hrs (n=)	45 hrs (n=)	55 hrs (n=)
wild type	**33.1 ± 1.8 (32)**	**51.5 ± 1.9 (20)**	**71.3 ± 1.9 (21)**	**17.0 ± 0.9 (21)**
let-418(n3536)	10.5 ± 1.0 (21)	10.6 ± 0.9 (25)	10.6 ± 0.7 (20)	10.8 ± 1.0 (22)
let-418(n3536) + LET-418 in intestine	**31.3 ± 2.2 (24)**	**36.2 ± 2.6 (21)**	**42.3 ± 6.7 (38)**	**25.1 ± 4.0 (30)**
let-418(n3536) + LET-418 in hypodermis	**32.2 ± 3.6 (37)**	**26.2 ± 3.4 (20)**	**15.5 ± 3.9 (38)**	**17.9 ± 2.0 (27)**
let-418(n3536) + LET-418 in muscles	10.4 ± 0.7 (33)	10.7 ± 0.9 (20)	10.7 ± 0.8 (21)	10.5 ± 0.7 (20)
let-418(n3536) + LET-418 in neurons	10.7 ± 1.2 (21)	10.7 ± 1.2 (23)	10.8 ± 1.2 (21)	10.7 ± 1.1 (25)
let-418(n3536) + LET-418 in germline	10.4 ± 0.7 (33)	10.4 ± 0.7 (21)	10.2 ± 0.4 (25)	10.6 ± 0.7 (25)
let-418(n3536) + LET-418 in M cell	10.4 ± 0.7 (21)	10.4 ± 0.7 (21)	10.4 ± 0.6 (23)	10.4 ± 0.6 (21)

Bold: p values <0.05

Supplemental Figure S3

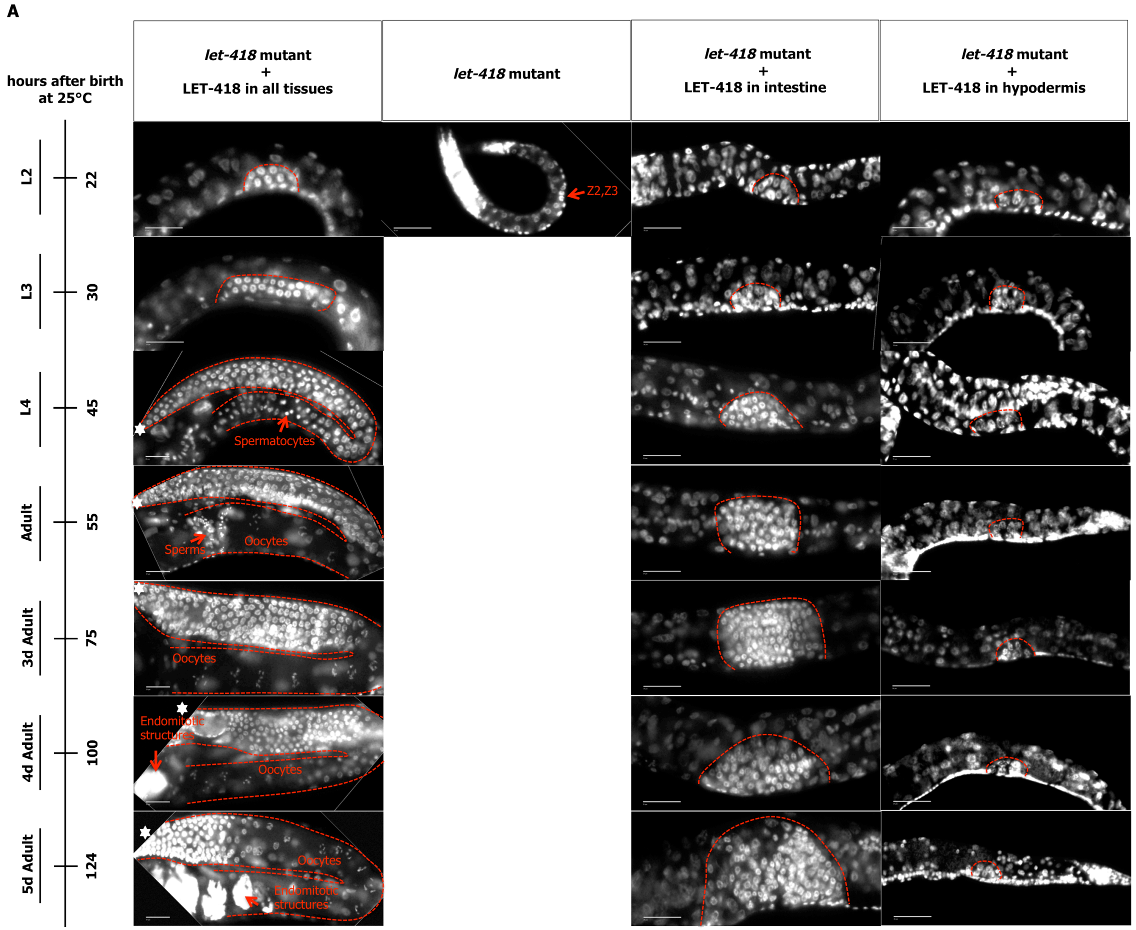

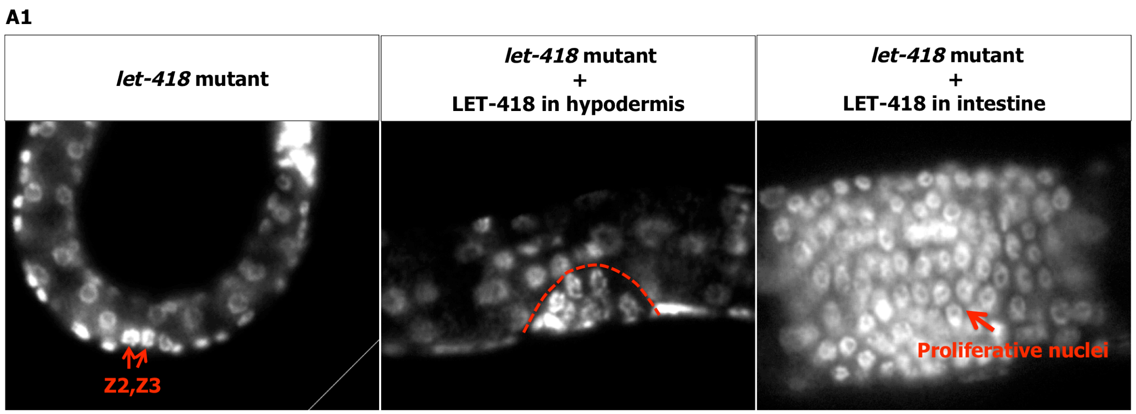

Sup Fig. 3. Germ cell proliferation and differentiation in worms expressing *let-418* in various tissues.

(**A**) DAPI-stained germline of *let-418(n3536)*, and LET-418/Mi-2 tissue-specific rescued lines. Germline development was followed during six days after birth. Germ cells were counted at 22, 30, 45, 55, 75, 100, 124 and 148 hours after birth. In *let-418* L1 larvae, including those with LET-418 expression in muscles, M cell, neurons, or PGCs, the Z2 and Z3 cells did not divide. *let-418(n3536)* mutants were completely rescued to the wild type level with LET-418/Mi-2 under its own promoter. Scale bar: 20 μm. **A1**. Worms with hypodermal LET-418/Mi-2 expression had undeveloped gonads and low levels of germ cell proliferation. Animals rescued with intestinal LET-418/Mi-2 expression had mainly unextended gonadal arm filled with proliferative nuclei.

Supplemental Table S5

Hours after birth (hrs)	Percentage of worms exhibiting 2-3 or more germ cells							
	Strains							
	let-418(n3536)	*let-418(n3536)* + LET-418 in all tissues	*let-418(n3536)* + LET-418 in intestine	*let-418(n3536)* + LET-418 in hypodermis	*let-418(n3536)* + LET-418 in muscles	*let-418(n3536)* + LET-418 in neurons	*let-418(n3536)* + LET-418 in germline	*let-418(n3536)* + LET-418 in M cell
22 hrs								
2 - 3 cells	100.0% (15)	-	-	22.5% (9)	100.0% (22)	100.0% (21)	100.0% (20)	100.0% (20)
>3 cells	-	**100.0% (11)**	**100.0% (20)**	**77.5% (31)**	-	-	-	-
30 hrs								
2 - 3 cells	100.0% (17)	-	-	7.4% (2)	100.0% (23)	100.0% (22)	100.0% (20)	100.0% (21)
>3 cells	-	**100.0% (13)**	**100.0% (20)**	**92.6 (25)**	-	-	-	-
45 hrs								
2 - 3 cells	100.0% (11)	-	-	-	100.0% (22)	100.0% (20)	100.0% (21)	100.0% (20)
>3 cells	-	**100.0% (15)**	**100.0% (28)**	**100.0% (41)**	-	-	-	-
55 hrs								
2 - 3 cells	100.0% (14)	-	-	-	100.0% (20)	100.0% (21)	100.0% (23)	100.0% (23)
>3 cells	-	**100.0% (5)**	**100.0% (20)**	**100.0% (20)**	-	-	-	-
75 hrs								
2 - 3 cells		-	-	2.7% (1)				
>3 cells		**100.0% (10)**	**100.0% (36)**	**97.3% (36)**				
100 hrs								
2 - 3 cells		-	-	-				
>3 cells		**100.0% (8)**	**100.0% (20)**	**100.0% (36)**				
124 hrs								
2 - 3 cells		-	-	-				
>3 cells		**100.0% (9)**	**100.0% (27)**	**100.0% (26)**				
148 hrs								
2 - 3 cells		-	-	-				
>3 cells		**100.0% (5)**	**100.0% (20)**	**100.0% (20)**				

Supplemental Table S6

	**Average number of germ cells**							
	Strains							
Hours after birth (hrs)	*let-418(n3536)*	*let-418(n3536)* + LET-418 in all tissues	*let-418(n3536)* + LET-418 in intestine	*let-418(n3536)* + LET-418 in hypodermis	*let-418(n3536)* + LET-418 in muscles	*let-418(n3536)* + LET-418 in neurons	*let-418(n3536)* + LET-418 in germline	*let-418(n3536)* + LET-418 in M cell
22 hrs								
undifferentiated germ cells	2.1 ± 0.2 (15)	**17.1 ± 1.3 (11)**	**14.0 ± 3.0 (20)**	**3.3 ± 1.0 (40)**	2.1 ± 0.3 (22)	2.0 ± 0.2 (21)	2.1 ± 0.2 (20)	2.1 ± 0.2 (20)
30 hrs								
undifferentiated germ cells	2.1 ± 0.2 (17)	**55.5 ± 2.3 (13)**	**27.1 ± 3.8 (20)**	**5.0 ± 1.2 (27)**	2.1 ± 0.3 (23)	2.0 ± 0.2 (22)	2.0 ± 0.0 (20)	2.0 ± 0.2 (21)
45 hrs								
undifferentiated germ cells	2.1 ± 0.3 (11)	**443.2 ± 8.3 (15)**	**55.4 ± 11.8 (28)**	**7.1 ± 1.4 (41)**	2.1 ± 0.3 (22)	2.1 ± 0.3 (20)	2.0 ± 0.2 (21)	2.0 ± 0.0 (20)
55 hrs								
undifferentiated germ cells	2.1 ± 0.3 (14)	**864.2 ± 9.2 (5)**	**79.5 ± 26.4 (20)**	**7.2 ±1.0 (20)**	2.1 ± 0.3 (20)	2.1 ± 0.3 (21)	2.0 ± 0.2 (23)	2.0 ± 0.2 (23)
sperms	-	268.4 ± 5.9 (5)	-	-	-	-	-	-
oocytes	-	9.8 ± 1.8 (5)	-	-	-	-	-	-
Total number of germ cells	2.1 (14)	1142.4 (5)	79.5 (20)	7.2 (20)	2.1 (20)	2.1 (21)	2.0 (23)	2.0 (23)
75 hrs								
undifferentiated germ cells		**1195.4 ± 17.1 (10)**	**93.6 ± 34 (36)**	**7.7 ± 2.1 (37)**				
sperms		32.6 ± 6.5 (10)	1.8 ± 6.0 (36)	-				
oocytes		19.3 ± 2.5 (10)	-	-				
Total number of germ cells		1247.3 (10)	95.4 (36)	7.7 (37)				
100 hrs								
undifferentiated germ cells		**1058.1 ± 21.6 (8)**	**104.7 ± 25.8 (20)**	**8.7 ± 2.2 (36)**				
sperms		2.0 ± 2.6 (8)	1.4 ± 3.8 (20)	-				
oocytes		28.8 ± 6.1 (8)	-	-				
Total number of germ cells		1088.9 (8)	106.1 (20)	8.7 (36)				
124 hrs								
undifferentiated germ cells		**1024.2 ± 24.2 (9)**	**123.6 ± 39.8 (27)**	**10.0 ± 2.6 (26)**				
sperms		-	1.0 ± 2.5 (27)	-				
oocytes		32.6 ± 8.4 (9)	-	-				
Total number of germ cells		1056.8 (9)	124.6 (27)	10.0 (26)				
148 hrs								
undifferentiated germ cells		**928.0 ± 25.2 (5)**	**95.0 ± 25.1 (20)**	**12.5 ± 2.2 (20)**				
sperms		-	0.2 ± 0.7 (20)	-				
oocytes		26.8 ± 6.6 (5)	-	-				
Total number of germ cells		954.8 (5)	95.1 (20)	12.5 (20)				

Bold: p values <0.05

Supplemental Table S7

Percentage of worms exhibiting 1-2 or more HLH-8::GFP positive cells								
Strains	Hours after birth (hrs)							
	22 hrs		30 hrs		45 hrs		55 hrs	
	1 or 2 cells	>2 cells	1 or 2 cells	>2 cells	1 or 2 cells	>2 cells	1 or 2 cells	>2 cells
wild type	-	**100.0% (20)**	-	**100.0% (23)**	-	**100% (20)**	33.3% (7)	**19.0% (4)**
let-418(n3536)	100.0% (21)	-	100.0% (21)	-	100.0% (23)	-	100% (22)	-
let-418(n3536) + LET-418 in intestine	100.0% (30)	-	67.6% (25)	**32.4% (12)**	58.8% (20)	**11.8% (4)**	32.6%(14)	**4.7% (2)**
let-418(n3536) + LET-418 in M cell	100.0% (33)	-	100.0% (30)	-	100.0% (21)	-	93.3% (28)	-
let-418(n3536) + LET-418 in intestine and M cell	73.3% (22)	**26.7% (8)**	21.7% (5)	**78.3% (18)**	22.7% (5)	**77.3% (17)**	4.8% (1)	**95.2% (20)**
let-418(n3536) + LET-418 in hypodermis	100.0% (22)	-	80.0% (20)	**20.0% (5)**	38.8% (19)	**10.2% (5)**	21.6% (8)	**2.7% (1)**
let-418(n3536) + LET-418 in intestine and hypodermis	100.0% (29)	-	38.1% (8)	**61.9% (13)**	55.3% (21)	**10.5% (4)**	25.0% (5)	**10.0% (2)**
let-418(n3536) + LET-418 in muscles	100.0% (22)	-	100.0% (35)	-	100.0%(30)	-	95.8% (23)	-
let-418(n3536) + LET-418 in intestine and muscles	96.0% (24)	**4.0% (1)**	75.0% (15)	**25.0% (5)**	26.1% (6)	**26.1% (6)**	30.0% (6)	**15.0% (3)**
let-418(n3536) + LET-418 in neurons	100.0% (27)	-	100.0% (22)	-	100.0% (21)	-	85.7% (18)	-
let-418(n3536) + LET-418 in intestine and neurons	100.0% (20)	-	75.0% (15)	**25.0% (5)**	40.0% (8)	**25.0% (5)**	42.9% (9)	**4.8% (1)**
let-418(n3536) + LET-418 in germline	100.0% (21)	-	100.0% (21)	-	100.0% (33)	-	90.9% (20)	-
let-418(n3536) + LET-418 in intestine and germline	96.3% (26)	**3.7% (1)**	76.2% (16)	**23.8% (5)**	28.6% (6)	**19.0% (4)**	35.0% (7)	-

Supplemental Table S8

Average number of HLH-8::GFP positive cells				
Strains	Hours after birth (hrs); number of worms (n=)			
	22 hrs (n=)	30 hrs (n=)	45 hrs (n=)	55 hrs (n=)
wild type	**16.3 ± 1.2 (20)**	**18.0 ± 0.0 (23)**	**16.0 ± 0.0 (20)**	1.3 ± 1.4 (21)
let-418(n3536)	1.1 ± 0.3 (21)	1.1 ± 0.3 (21)	1.1 ± 0.3 (23)	1.1 ± 0.3 (22)
let-418(n3536) + LET-418 in intestine	1.1 ± 0.3 (30)	**2.4 ± 1.8 (37)**	1.4 ± 1.8 (34)	0.6 ± 0.9 (43)
let-418(n3536) + LET-418 in M cell	1.2 ± 0.4 (33)	1.4 ± 0.5 (30)	1.2 ± 0.4 (21)	1.2 ± 0.5 (30)
let-418(n3536) + LET-418 in intestine and M cell	**2.3 ± 1.8 (30)**	**9.1 ± 5.6 (23)**	**5.8 ± 3.3 (22)**	**5.7 ± 1.5 (21)**
let-418(n3536) + LET-418 in hypodermis	1.2 ± 0.4 (22)	**2.2 ± 2.0 (25)**	0.8 ± 1.1 (49)	0.4 ± 0.8 (37)
let-418(n3536) + LET-418 in intestine and hypodermis	1.2 ± 0.4 (29)	**2.6 ± 1.2 (21)**	1.3 ± 1.7 (38)	0.8 ± 1.3 (20)
let-418(n3536) + LET-418 in muscles	1.1 ± 0.3 (22)	1.1 ± 0.3 (35)	1.0 ± 0.2 (30)	1.0 ± 0.4 (24)
let-418(n3536) + LET-418 in intestine and muscles	1.6 ± 0.7 (25)	**2.3 ± 1.6 (20)**	1.7 ± 2.6 (23)	1.0 ± 1.2 (20)
let-418(n3536) + LET-418 in neurons	1.1 ± 0.3 (27)	1.0 ± 0.2 (22)	1.0 ± 0.2 (21)	1.0 ± 0.5 (21)
let-418(n3536) + LET-418 in intestine and neurons	1.2 ± 0.4 (20)	**2.0 ± 1.0 (20)**	1.8 ± 2.2 (20)	0.8 ± 0.9 (21)
let-418(n3536) + LET-418 in germline	1.1 ± 0.3 (21)	1.1 ± 0.3 (21)	1.1 ± 0.3 (33)	1.1 ± 0.5 (22)
let-418(n3536) + LET-418 in intestine and germline	1.5 ± 0.6 (27)	**2.0 ± 0.9 (21)**	1.4 ± 2.1 (21)	0.4 ± 0.6 (20)

Bold: p values <0.05

Supplemental Table S9

Percentage of worms exhibiting 1-2 or more HLH-8::GFP positive cells								
Strains	Hours after birth (hrs)							
	22 hrs		30 hrs		45 hrs		55 hrs	
	1 or 2 cells	>2 cells	1 or 2 cells	>2 cells	1 or 2 cells	>2 cells	1 or 2 cells	>2 cells
wild type	-	**100.0% (20)**	-	**100.0% (23)**	-	**100% (20)**	33.3% (7)	**19.0% (4)**
let-418(n3536)	100.0% (21)	-	100.0% (21)	-	100.0% (23)	-	100% (22)	-
let-418(n3536) + LET-418 in hypodermis	100.0% (22)	-	80.0% (20)	**20.0% (5)**	38.8% (19)	**10.2% (5)**	21.6% (8)	**2.7% (1)**
let-418(n3536) + LET-418 in M cell	100.0% (33)	-	100.0% (30)	-	100.0% (21)	-	93.3% (28)	-
let-418(n3536) + LET-418 in hypodermis and M cell	82.8% (24)	**17.2% (5)**	9.5% (2)	**90.5% (19)**	33.3% (8)	**66.7% (16)**	23.8% (5)	**71.4% (15)**
let-418(n3536) + LET-418 in intestine	100.0% (30)	-	67.6% (25)	**32.4% (12)**	58.8% (20)	**11.8% (4)**	32.6%(14)	**4.7% (2)**
let-418(n3536) + LET-418 in hypodermis and intestine	100.0% (29)	-	38.1% (8)	**61.9% (13)**	55.3% (21)	**10.5% (4)**	25.0% (5)	**10.0% (2)**
let-418(n3536) + LET-418 in muscles	100.0% (22)	-	100.0% (35)	-	100.0%(30)	-	95.8% (23)	-
let-418(n3536) + LET-418 in hypodermis and muscles	94.6% (35)	**5.4% (2)**	52.4% (11)	**47.6% (10)**	52.4% (22)	**2.4% (1)**	10.0% (2)	**10.0% (2)**
let-418(n3536) + LET-418 in neurons	100.0% (27)	-	100.0% (22)	-	100.0% (21)	-	85.7% (18)	-
let-418(n3536) + LET-418 in hypodermis and neurons	100.0% (24)	-	72.0% (18)	**28.0% (7)**	56.5% (13)	**4.3% (1)**	36.7% (11)	-
let-418(n3536) + LET-418 in germline	100.0% (21)	-	100.0% (21)	-	100.0% (33)	-	90.9% (20)	-
let-418(n3536) + LET-418 in hypodermis and germline	96.9% (31)	**3.1% (1)**	75.0% (15)	**25.0% (5)**	50.0% (10)	**5.0% (1)**	23.8% (5)	-

Supplemental Table S10

Average number of HLH-8::GFP positive cells				
Strains	Hours after birth (hrs); number of worms (n=)			
	22 hrs (n=)	30 hrs (n=)	45 hrs (n=)	55 hrs (n=)
wild type	**16.3 ± 1.2 (20)**	**18.0 ± 0.0 (23)**	**16.0 ± 0.0 (20)**	1.3 ± 1.4 (21)
let-418(n3536)	1.1 ± 0.3 (21)	1.1 ± 0.3 (21)	1.1 ± 0.3 (23)	1.1 ± 0.3 (22)
let-418(n3536) + LET-418 in hypodermis	1.2 ± 0.4 (22)	**2.2 ± 2.0 (25)**	0.8 ± 1.1 (49)	0.4 ± 0.8 (37)
let-418(n3536) + LET-418 in M cell	1.2 ± 0.4 (33)	1.4 ± 0.5 (30)	1.2 ± 0.4 (21)	1.2 ± 0.5 (30)
let-418(n3536) + LET-418 in hypodermis and M cell	1.9 ± 1.0 (29)	**9.7 ± 4.4 (21)**	**3.8 ± 2.3 (24)**	**4.0 ± 2.2 (21)**
let-418(n3536) + LET-418 in intestine	1.1 ± 0.3 (30)	**2.4 ± 1.8 (37)**	1.4 ± 1.8 (34)	0.6 ± 0.9 (43)
let-418(n3536) + LET-418 in hypodermis and intestine	1.2 ± 0.4 (29)	**2.6 ± 1.2 (21)**	1.3 ± 1.7 (38)	0.8 ± 1.3 (20)
let-418(n3536) + LET-418 in muscles	1.1 ± 0.3 (22)	1.1 ± 0.3 (35)	1.0 ± 0.2 (30)	1.0 ± 0.4 (24)
let-418(n3536) + LET-418 in hypodermis and muscles	1.3 ± 0.9 (37)	**2.3 ± 1.1 (21)**	0.7 ± 0.8 (42)	0.5 ± 1.0 (20)
let-418(n3536) + LET-418 in neurons	1.1 ± 0.3 (27)	1.0 ± 0.2 (22)	1.0 ± 0.2 (21)	1.0 ± 0.5 (21)
let-418(n3536) + LET-418 in hypodermis and neurons	1.3 ± 0.4 (24)	1.9 ± 1.3 (25)	1.0 ± 1.0 (23)	0.4 ± 0.6 (30)
let-418(n3536) + LET-418 in germline	1.1 ± 0.3 (21)	1.1 ± 0.3 (21)	1.1 ± 0.3 (33)	1.1 ± 0.5 (22)
let-418(n3536) + LET-418 in hypodermis and germline	1.4 ± 0.6 (32)	1.9 ± 1.2 (20)	0.8 ± 0.8 (20)	0.2 ± 0.4 (21)

Bold: p values <0.05

Supplemental Figure S4

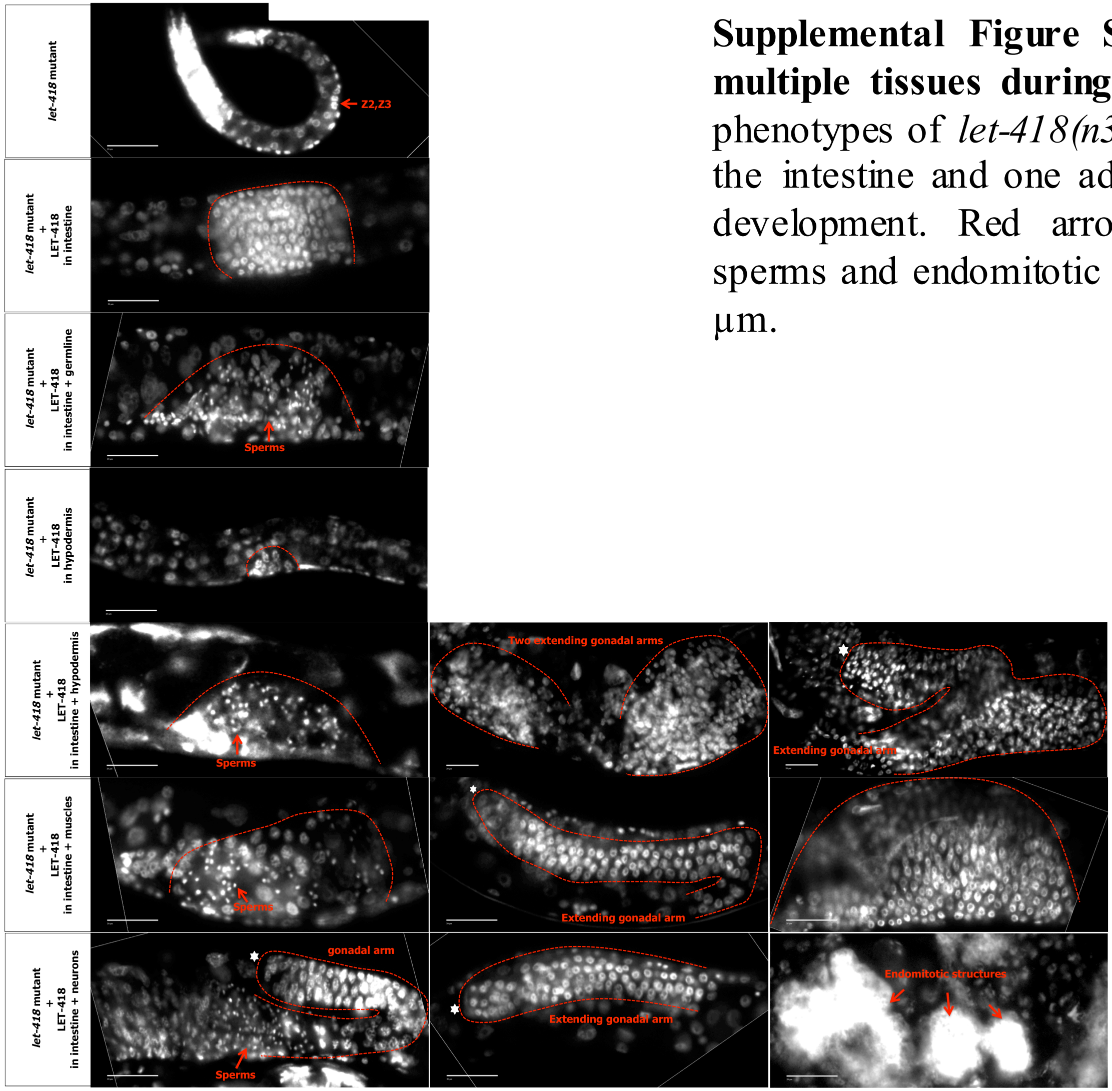

Supplemental Figure S4. LET-418/Mi-2 is required in multiple tissues during germline development. Germline phenotypes of *let-418(n3536)* animals expressing *let-418* in the intestine and one additional tissue at terminal stage of development. Red arrows are pointing towards Z2/Z3, sperms and endomitotic oocytes as indicated. Scale bar: 20 µm.

Supplemental Table S11

**Percentage of worms with sperms**											
	Strains										
Hours after birth (hrs)	*let-418(n3536)*	*let-418(n3536)* + LET-418 in all tissues	*let-418(n3536)* + LET-418 in intestine	*let-418(n3536)* + LET-418 in germline	*let-418(n3536)* + LET-418 in intestine and germline	*let-418(n3536)* + LET-418 in hypodermis	*let-418(n3536)* + LET-418 in intestine and hypodermis	*let-418(n3536)* + LET-418 in muscles	*let-418(n3536)* + LET-418 in intestine and muscles	*let-418(n3536)* + LET-418 in neurons	*let-418(n3536)* + LET-418 in intestine and neurons
22 hrs											
worms with sperms	-	-	-	-	-	-	-	-	-	-	-
worms without sperms	100% (15)	100% (11)	100% (20)	100% (20)	100% (23)	100% (40)	100% (21)	100% (22)	100% (21)	100% (21)	100% (21)
30 hrs											
worms with sperms	-	-	-	-	-	-	-	-	-	-	-
worms without sperms	100% (17)	100% (13)	100% (20)	100% (20)	100% (21)	100% (27)	100% (23)	100% (23)	100% (21)	100% (22)	100% (23)
45 hrs											
worms with sperms	-	-	-	-	-	-	-	-	-	-	-
worms without sperms	100% (11)	100% (15)	100% (28)	100% (21)	100% (29)	100% (41)	100% (22)	100% (22)	100% (30)	100% (20)	100% (25)
55 hrs											
worms with sperms	-	**100% (5)**	-	-	-	-	-	-	-	-	-
worms without sperms	100% (14)	-	100% (20)	100% (23)	100% (22)	100% (20)	100% (20)	100% (20)	100% (20)	100% (21)	100% (36)
75 hrs											
worms with sperms		**100% (10)**	**11% (4)**		**55% (11)**	-	**45% (9)**		**30% (6)**		**19% (6)**
worms without sperms		-	89% (32)		45% (9)	100% (37)	55% (11)		70% (14)		81% (25)
100 hrs											
worms with sperms		**37.5% (3)**	**15% (3)**		**45% (9)**	-	**48% (10)**		**19% (5)**		**13% (4)**
worms without sperms		62.5% (5)	85% (17)		55% (11)	100% (36)	52% (11)		81% (21)		87% (26)
124 hrs											
worms with sperms		-	**15% (4)**		**42% (11)**	-	**40% (8)**		**27% (6)**		**24% (5)**
worms without sperms		100% (9)	85% (23)		58% (15)	100% (26)	60% (12)		73% (16)		76% (16)
148 hrs											
worms with sperms		-	**5% (1)**		**43% (9)**	-	**40% (8)**		**25% (5)**		**25% (5)**
worms without sperms		100% (5)	95% (19)		57% (12)	100% (20)	60% (12)		75% (15)		75% (15)

Supplemental Table S12

Average number of germ cells											
Hours after birth (hrs)	Strains										
	let-418(n3536)	*let-418(n3536)* + LET-418 in all tissues	*let-418(n3536)* + LET-418 in intestine	*let-418(n3536)* + LET-418 in germline	*let-418(n3536)* + LET-418 in intestine and germline	*let-418(n3536)* + LET-418 in hypodermis	*let-418(n3536)* + LET-418 in intestine and hypodermis	*let-418(n3536)* + LET-418 in muscles	*let-418(n3536)* + LET-418 in intestine and muscles	*let-418(n3536)* + LET-418 in neurons	*let-418(n3536)* + LET-418 in intestine and neurons
22 hrs											
undifferentiated germ cells	2.1 ± 0.2 (15)	**17.1 ± 1.3 (11)**	**14.0 ± 3.0 (20)**	2.1 ± 0.2 (20)	**14.5 ± 2.1 (23)**	**3.3 ± 1.0 (40)**	**11.3 ± 2.1 (21)**	2.1 ± 0.3 (22)	**12.0 ± 3.0 (21)**	2.0 ± 0.2 (21)	**14.2 ± 2.4 (21)**
30 hrs											
undifferentiated germ cells	2.1 ± 0.2 (17)	**55.5 ± 2.3 (13)**	**27.1 ±3.8 (20)**	2.0 ± 0.0 (20)	**29.0 ± 3.1 (21)**	**5.0 ± 1.2 (27)**	**26.3 ± 3.9 (23)**	2.1 ± 0.3 (23)	**25.0 ± 4.0 (21)**	2.0 ± 0.2 (22)	**26.9 ± 3.9 (23)**
45 hrs											
undifferentiated germ cells	2.1 ± 0.3 (11)	**443.2 ± 8.3 (15)**	**55.4 ± 11.8 (28)**	2.0 ± 0.2 (21)	**57.0 ± 10.2 (29)**	**7.1 ± 1.4 (41)**	**51.5 ± 5.7 (22)**	2.1 ± 0.3 (22)	**53.4 ± 13.7 (30)**	2.1 ± 0.3 (20)	**59.0 ± 8.8 (25)**
55 hrs											
undifferentiated germ cells	2.1 ± 0.3 (14)	**864.2 ± 9.2 (5)**	**79.5 ± 26.4 (20)**	2.0 ± 0.2 (23)	**71.2 ± 11.3 (22)**	**7.2 ± 1.0 (20)**	**78.0 ± 22.7 (20)**	2.1 ± 0.3 (20)	**73.7 ± 16.0 (20)**	2.1 ± 0.3 (21)	**101.1 ± 21.6 (36)**
sperms	-	268.4 ± 5.9 (5)	-	-	-	-	-	-	-	-	-
oocytes	-	9.8 ± 1.8 (5)	-	-	-	-	-	-	-	-	-
Total number of germ cells	2.1 (14)	1142.4 (5)	79.5 (20)	2.0 (23)	71.2 (22)	7.2 (20)	78.0 (20)	2.1 (20)	73.7 (20)	2.1 (21)	101.1 (36)
75 hrs											
undifferentiated germ cells		**1195.4 ± 17.1 (10)**	**93.6 ± 34 (36)**		**78.4 ± 34.7 (20)**	**7.7 ± 2.1 (37)**	**80.9 ± 24.0 (20)**		**84.3 ± 36.4 (20)**		**125.8 ± 67.7 (31)**
sperms		32.6 ± 6.5 (10)	1.8 ± 6.0 (36)		17.9 ± 22.0 (20)	-	20.4 ± 31.9 (20)		17.6 ± 28.3 (20)		12.3 ± 35.4 (31)
oocytes		19.3 ± 2.5 (10)	-		-	-	-		-		-
Total number of germ cells		1247.3 (10)	95.4 (36)		96.3 (20)	7.7 (37)	101.3 (20)		101.9 (20)		138.1 (31)
100 hrs											
undifferentiated germ cells		**1058.1 ± 21.6 (8)**	**104.7 ± 25.8 (20)**		**111.85 ± 26.9 (20)**	**8.7 ± 2.2 (36)**	**102.8 ± 69.6 (21)**		**96.9 ± 34.1 (26)**		**129.8 ± 58.4 (30)**
sperms		2.0 ± 2.6 (8)	1.4 ± 3.8 (20)		18.05 ± 27.7 (20)	-	21.1 ± 25.1 (21)		13.3 ± 28.8 (26)		12.8 ± 35.9 (30)
oocytes		28.8 ± 6.1 (8)	-		-	-	-		-		-
Total number of germ cells		1088.9 (8)	106.1 (20)		129.9 (20)	8.7 (36)	123.9 (21)		110.2 (26)		142.6 (30)
124 hrs											
undifferentiated germ cells		**1024.2 ± 24.2 (9)**	**123.6 ± 39.8 (27)**		**95.15 ± 36.5 (26)**	**10.0 ± 2.6 (26)**	**105.0 ± 52.6 (20)**		**102.4 ± 25.3 (22)**		**145.7 ± 41.5 (21)**
sperms		-	1.0 ± 2.5 (27)		17.08 ± 26.9 (26)	-	27.9 ± 39.7 (20)		12.0 ± 20.3 (22)		11.9 ± 22.7 (21)
oocytes		32.6 ± 8.4 (9)	-		-	-	-		-		-
Total number of germ cells		1056.8 (9)	124.6 (27)		112.2 (26)	10.0 (26)	132.9 (20)		114.4 (22)		157.6 (21)
148 hrs											
undifferentiated germ cells		**928.0 ± 25.2 (5)**	**95.0 ± 25.1 (20)**		**93.3 ± 23.0 (21)**	**12.5 ± 2.2 (20)**	**110.1 ± 52.7 (20)**		**115.0 ± 65.9 (20)**		**175.75 ± 86.4 (20)**
sperms		-	0.2 ± 0.7 (20)		12.5 ± 18.3 (21)	-	21.2 ± 35.7 (20)		11.8 ± 23.2 (20)		12.35 ± 23.0 (20)
oocytes		26.8 ± 6.6 (5)	-		-	-	-		-		-
Total number of germ cells		954.8 (5)	95.1 (20)		105.8 (21)	12.5 (20)	131.3 (20)		126.8 (20)		188.1 (20)

Bold: p values <0.05

Supplemental Table S13

Average number of germ cells											
	Strains										
Hours after birth (hrs)	*let-418(n3536)*	*let-418(n3536)* + LET-418 in all tissues	*let-418(n3536)* + LET-418 in hypodermis	*let-418(n3536)* + LET-418 in germline	*let-418(n3536)* + LET-418 in hypodermis and germline	*let-418(n3536)* + LET-418 in intestine	*let-418(n3536)* + LET-418 in hypodermis and intestine	*let-418(n3536)* + LET-418 in muscles	*let-418(n3536)* + LET-418 in hypodermis and muscles	*let-418(n3536)* + LET-418 in neurons	*let-418(n3536)* + LET-418 in hypodermis and neurons
22 hrs											
undifferentiated germ cells	2.1 ± 0.2 (15)	**17.1 ± 1.3 (11)**	**3.3 ± 1.0 (40)**	2.1 ± 0.2 (20)	**3.7 ± 1.1 (23)**	**14.0 ± 3.0 (20)**	**11.3 ± 2.1 (21)**	2.1 ± 0.3 (22)	**3.8 ± 0.9 (20)**	2.0 ± 0.2 (21)	**3.9 ± 1.1 (23)**
30 hrs											
undifferentiated germ cells	2.1 ± 0.2 (17)	**55.5 ± 2.3 (13)**	**5.0 ± 1.2 (27)**	2.0 ± 0.0 (20)	**5.3 ± 1.7 (21)**	**27.1 ± 3.8 (20)**	**26.3 ± 3.9 (23)**	2.1 ± 0.3 (23)	**5.7 ± 1.2 (21)**	2.0 ± 0.2 (22)	**5.2 ± 1.6 (21)**
45 hrs											
undifferentiated germ cells	2.1 ± 0.3 (11)	**443.2 ± 8.3 (15)**	**7.1 ± 1.4 (41)**	2.0 ± 0.2 (21)	**7.2 ± 1.4 (23)**	**55.4 ± 11.8 (28)**	**51.5 ± 5.7 (22)**	2.1 ± 0.3 (22)	**6.5 ± 1.7 (21)**	2.1 ± 0.3 (20)	**7.3 ± 1.4 (22)**
55 hrs											
undifferentiated germ cells	2.1 ± 0.3 (14)	**864.2 ± 9.2 (5)**	**7.2 ± 1.0 (20)**	2.0 ± 0.2 (23)	**7.6 ± 1.3 (23)**	**79.5 ± 26.4 (20)**	**78.0 ± 22.7 (20)**	2.1 ± 0.3 (20)	**7.3 ± 1.0 (21)**	2.1 ± 0.3 (21)	**7.4 ± 1.7 (20)**
sperms	-	268.4 ± 5.9 (5)	-	-	-	-	-	-	-	-	-
oocytes	-	9.8 ± 1.8 (5)	-	-	-	-	-	-	-	-	-
Total number of germ cells	2.1 (14)	1142.4 (5)	7.2 (20)	2.0 (23)	7.6 (23)	79.5 (20)	78.0 (20)	2.1 (20)	7.3 (21)	2.1 (21)	7.4 (20)
75 hrs											
undifferentiated germ cells		**1195.4 ± 17.1 (10)**	**7.7 ± 2.1 (37)**		**7.9 ± 1.8 (21)**	**93.6 ± 34.0 (36)**	**80.9 ± 24.0 (20)**		**8.2 ± 2.2 (21)**		**7.8 ± 1.9 (22)**
sperms		32.6 ± 6.5 (10)	-		-	1.8 ± 6.0 (36)	20.4 ± 31.9 (20)		-		-
oocytes		19.3 ± 2.5 (10)	-		-	-	-		-		-
Total number of germ cells		1247.3 (10)	7.7 (37)		7.9 (21)	95.4 (36)	101.3 (20)		8.2 (21)		7.8 (22)
100 hrs											
undifferentiated germ cells		**1058.1 ± 21.6 (8)**	**8.7 ± 2.2 (36)**		**9.5 ± 2.6 (24)**	**104.7 ± 25.8 (20)**	**102.8 ± 69.6 (21)**		**9.8 ± 1.9 (21)**		**9.3 ± 2.4 (22)**
sperms		2.0 ± 2.6 (8)	-		-	1.4 ± 3.8 (20)	21.1 ± 25.1 (21)		-		-
oocytes		28.8 ± 6.1 (8)	-		-	-	-		-		-
Total number of germ cells		1088.9 (8)	8.7 (36)		9.5 (24)	106.1 (20)	123.9 (21)		9.8 (21)		9.3 (22)
124 hrs											
undifferentiated germ cells		**1024.2 ± 24.2 (9)**	**10.0 ± 2.6 (26)**		**9.8 ± 2.6 (25)**	**123.6 ± 39.8 (27)**	**105.0 ± 52.6 (20)**		**10.1 ± 3.0 (20)**		**10.0 ± 2.5 (21)**
sperms		-	-		-	1.0 ± 2.5 (27)	27.9 ± 39.7 (20)		-		-
oocytes		32.6 ± 8.4 (9)	-		-	-	-		-		-
Total number of germ cells		1056.8 (9)	10.0 (26)		9.8 (25)	124.6 (27)	132.9 (20)		10.1 (20)		10.0 (21)
148 hrs											
undifferentiated germ cells		**928.0 ± 25.2 (5)**	**12.5 ± 2.2 (20)**		**11.6 ± 2.9 (21)**	**95.0 ± 25.1 (20)**	**110.1 ± 52.7 (20)**		**12.3 ± 2.8 (20)**		**11.9 ± 2.3 (20)**
sperms		-	-		-	0.2 ± 0.7 (20)	21.2 ± 35.7 (20)		-		-
oocytes		26.8 ± 6.6 (5)	-		-	-	-		-		-
Total number of germ cells		954.8 (5)	12.5 (20)		11.6 (21)	95.1 (20)	131.3 (20)		12.3 (20)		11.9 (20)

Bold: p values <0.05

Supplemental Figure S5

Supplemental Figure S5. Insulin signaling is required for the cell-nonautonomous function of LET-418 on M cell disvision. Representative pictures are shown for the indicated genetic background. Undifferentiated cell descendant of the M lineage were visualized using *hlh-8::gfp* reporter in 22, 30, 45 and 55 hours after birth. During postembryonic development of wild type worms, M mesoblast undergoes a series of divisions giving rise to BWMs, CCs and sex muscles. Scale bar: 20 µm.

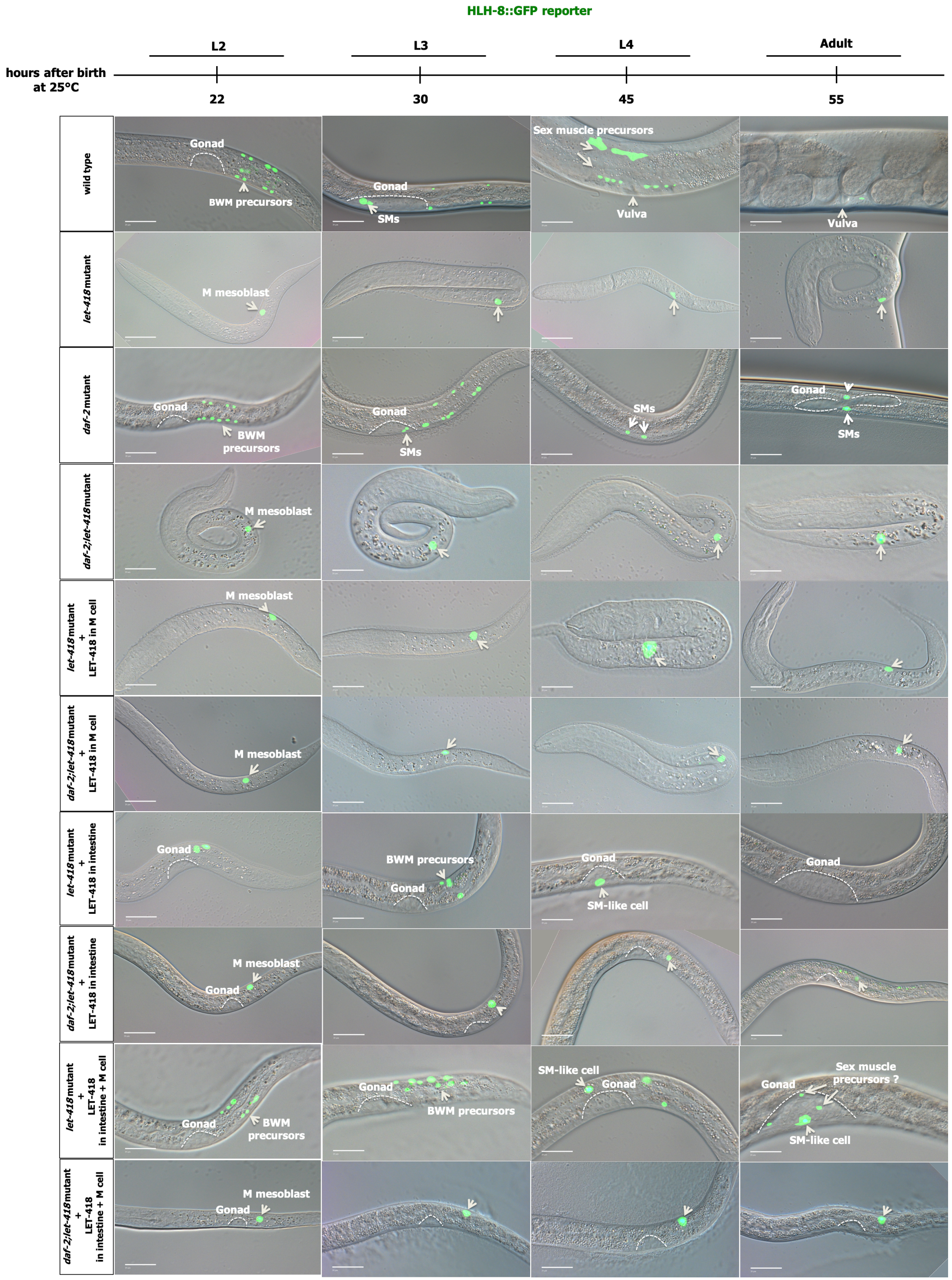

Supplemental Table S14

**Percentage of worms exhibiting 1-2 or more HLH-8::GFP positive cells**								
Strains	Hours after birth (hrs)							
	22 hrs		30 hrs		45 hrs		55 hrs	
	1 or 2 cells	>2 cells	1 or 2 cells	>2 cells	1 or 2 cells	>2 cells	1 or 2 cells	>2 cells
wild type	-	**100.0% (20)**	-	**100.0% (23)**	-	**100% (20)**	33.3% (7)	**19.0% (4)**
let-418(n3536)	100.0% (21)	-	100.0% (21)	-	100.0% (23)	-	100.0% (22)	-
daf-2(e1370)	-	**100.0% (22)**	-	**100% (21)**	95.2% (20)	**4.8% (1)**	81.0% (17)	**19.0% (4)**
daf-2(e1370);let-418(n3536)	100.0% (23)	-	100.0% (25)	-	100.0% (21)	-	100.0% (20)	-
let-418(n3536) + LET-418 in intestine	100.0% (30)	-	67.6% (25)	**32.4% (12)**	58.8% (20)	**11.8% (4)**	32.6%(14)	**4.7% (2)**
daf-2(e1370);let-418(n3536) + LET-418 in intestine	100.0% (38)	-	100.0% (21)	-	92.0% (23)	-	61.9% (13)	-
let-418(n3536) + LET-418 in M cell	100.0% (33)	-	100.0% (30)	-	100.0% (21)	-	93.3% (28)	-
daf-2(e1370);let-418(n3536) + LET-418 in M cell	100.0% (20)	-	100.0% (22)	-	100.0% (25)	-	95.7% (22)	-
let-418(n3536) + LET-418 in intestine and M cell	73.3% (22)	**26.7% (8)**	21.7% (5)	**78.3% (18)**	22.7% (5)	**77.3% (17)**	4.8% (1)	**95.2% (20)**
daf-2(e1370);let-418(n3536) + LET-418 in intestine and M cell	100.0% (27)	-	92.6% (25)	**7.4% (2)**	95.2% (20)	**4.8% (1)**	95.2% (20)	-

Supplemental Table S15

Average number of HLH-8::GFP positive cells				
Strains	Hours after birth (hrs); number of worms (n=)			
	22 hrs (n=)	30 hrs (n=)	45 hrs (n=)	55 hrs (n=)
wild type	**16.3 ± 1.2 (20)**	**18.0 ± 0.0 (23)**	**16.0 ± 0.0 (20)**	1.3 ± 1.4 (21)
let-418(n3536)	1.1 ± 0.3 (21)	1.1 ± 0.3 (21)	1.1 ± 0.3 (23)	1.1 ± 0.3 (22)
daf-2(e1370)	**11.3 ± 3.5 (22)**	**17.1 ± 1.2 (21)**	2.0 ± 0.3 (21)	1.9 ± 0.7 (21)
daf-2(e1370);let-418(n3536)	1.0 ± 0.0 (23)	1.0 ± 0.0 (25)	1.0 ± 0.0 (21)	1.0 ± 0.0 (20)
let-418(n3536) + LET-418 in intestine	1.1 ± 0.3 (30)	**2.4 ± 1.8 (37)**	1.4 ± 1.8 (34)	0.6 ± 0.9 (43)
daf-2(e1370);let-418(n3536) + LET-418 in intestine	1.0 ± 0.0 (38)	1.0 ± 0.0 (21)	0.9 ± 0.3 (25)	0.6 ± 0.5 (21)
let-418(n3536) + LET-418 in M cell	1.2 ± 0.4 (33)	1.4 ± 0.5 (30)	1.2 ± 0.4 (21)	1.2 ± 0.5 (30)
daf-2(e1370);let-418(n3536) + LET-418 in M cell	1.0 ± 0.0 (20)	1.0 ± 0.0 (22)	1.0 ± 0.0 (25)	1.0 ± 0.2 (23)
let-418(n3536) + LET-418 in intestine and M cell	**2.3 ± 1.8 (30)**	**9.1 ± 5.6 (23)**	**5.8 ± 3.3 (25)**	**5.7 ± 1.5 (21)**
daf-2(e1370);let-418(n3536) + LET-418 in intestine and M cell	1.1 ± 0.3 (27)	1.2 ± 0.6 (27)	1.2 ± 0.5 (21)	1.0 ± 0.4 (21)

Bold: p values <0.05
