# Supplementary material for: The Chromatin Remodeler LET-418/Mi2 is Required Cell Non-Autonomously for the Post-Embryonic Development of Caenorhabditis elegans
Source: J Dev Biol. 2018 Dec 24;7(1):1. doi: 10.3390/jdb7010001 (PMC6473691; doi:10.3390/jdb7010001)
Supplement: Supplementary file 1 [file jdb-07-00001-s001.zip › jdb-399122-SI/Sup materials.pdf]

Supplementary materials

The following strains were used in this study:

Strain	Genotype
N2 (Bristol)	*C. elegans* wild-type
CB1370	*daf-2(e1370)* III
JR667	*unc-119(e2498::Tc1)* III; *wIs51[pMF1(scmp::gfp) + pDP#MM016b(unc-119p::unc-119(+)]* V
PD4666	*ayIs6[pBH47(hlh-8p::gfp) + dpy-20(+)] X*
EG8081	*unc-119(ed3)* III; *oxTi177[ttTi5605 + NeoR(+) + unc-18(+)]* IV
FR1382	*ttTi4348* I; *unc-119(ed3)* III
FR843	*let-418(n3536)* V
FR1007	*daf-2(e1370)* III; *let-418(n3536)* V
FR1209	*ayIs6[pBH47(hlh-8p::gfp) + dpy-20(+)] X*; *let-418(n3536)* V
FR1353	*Si3[let-418p::let-418ORF::3xFLAG::let-418 3'UTR + cb-unc-119(+)]* II; *unc-119(ed3)* III
FR1355	*Si3[let-418p::let-418ORF::3xFLAG::let-418 3'UTR + cb-unc-119(+)]* II; *unc-119(ed3)* III (?); *let-418(n3536)* V
FR1389	*Si10[pie-1p::let-418ORF::3xFLAG::let-418 3'UTR + cb-unc-119(+)]* I; *unc-119(ed3)* III
FR1392	*Si11[dpy-7p::let-418ORF::3xFLAG::let-418 3'UTR + cb-unc-119(+)]* I; *unc-119(ed3)* III
FR1393	*Si12[myo-3p::let-418ORF::3xFLAG::let-418 3'UTR + cb-unc-119(+)]* I; *unc-119(ed3)* III
FR1394	*Si12[myo-3p::let-418ORF::3xFLAG::let-418 3'UTR + cb-unc-119(+)]* I; *unc-119(ed3)* III (?); *let-418(n3536)* V
FR1395	*Si13[elt-2p::let-418ORF::3xFLAG::let-418 3'UTR + cb-unc-119(+)]* I; *unc-119(ed3)* III
FR1396	*Si13[elt-2p::let-418ORF::3xFLAG::let-418 3'UTR + cb-unc-119(+)]* I; *unc-119(ed3)* III (?); *let-418(n3536)* V
FR1399	*Si10[pie-1p::let-418ORF::3xFLAG::let-418 3'UTR + cb-unc-119(+)]* I; *unc-119(ed3)* III (?); *let-418(n3536)* V
FR1403	*Si11[dpy-7p::let-418ORF::3xFLAG::let-418 3'UTR + cb-unc-119(+)]* I; *unc-119(ed3)* III (?); *let-418(n3536)* V
FR1425	*Si13[elt-2p::let-418ORF::3xFLAG::let-418 3'UTR + cb-unc-119(+)]* I; *unc-119(ed3)* III (?); *let-418(n3536)* V; *ayIs6[pBH47(hlh-8p::gfp) + dpy-20(+)]* X
FR1427	*wIs51[pMF1(scmp::gfp) + pDP#MM016b(unc-119p::unc-119(+)]* V, *let-418(n3536)* V
FR1458	*Si13[elt-2p::let-418ORF::3xFLAG::let-418 3'UTR + cb-unc-119(+)]* I; *unc-119(ed3)* III (?); *wIs51[pMF1(scmp::gfp) + pDP#MM016b(unc-119p::unc-119(+)]* V, *let-418(n3536)* V
FR1459	*Si11[dpy-7p::let-418ORF::3xFLAG::let-418 3'UTR + cb-unc-119(+)]* I; *unc-119(ed3)* III (?); *let-418(n3536)* V; *ayIs6[pBH47(hlh-8p::gfp) + dpy-20(+)]* X
FR1460	*Si11[dpy-7p::let-418ORF::3xFLAG::let-418 3'UTR + cb-unc-119(+)]* I; *unc-119(ed3)* III (?); *wIs51[pMF1(scmp::gfp) + pDP#MM016b(unc-119p::unc-119(+)]* V, *let-418(n3536)* V
FR1461	*Si12[myo-3p::let-418ORF::3xFLAG::let-418 3'UTR + cb-unc-119(+)]* I; *unc-119(ed3)* III (?); *let-418(n3536)* V; *ayIs6[pBH47(hlh-8p::gfp) + dpy-20(+)]* X
FR1462	*Si12[myo-3p::let-418ORF::3xFLAG::let-418 3'UTR + cb-unc-119(+)]* I; *unc-119(ed3)* III (?); *wIs51[pMF1(scmp::gfp) + pDP#MM016b(unc-119p::unc-119(+)]* V, *let-418(n3536)* V;
FR1463	*Si10[pie-1p::let-418ORF::3xFLAG::let-418 3'UTR + cb-unc-119(+)]* I; *unc-119(ed3)* III (?); *let-418(n3536)* V; *ayIs6[pBH47(hlh-8p::gfp) + dpy-20(+)]* X
FR1464	*Si10[pie-1p::let-418ORF::3xFLAG::let-418 3'UTR + cb-unc-119(+)]* I; *unc-119(ed3)* III (?); *wIs51[pMF1(scmp::gfp) + pDP#MM016b(unc-119p::unc-119(+)]* V, *let-418(n3536)* V
FR1468	*Si18[hlh-8p::let-418ORF::3xFLAG::let-418 3'UTR + cb-unc-119(+)]* I; *unc-119(ed3)* III
FR1475	*Si22[rgef-1p::let-418ORF::3xFLAG::let-418 3'UTR + cb-unc-119(+)]* IV; *unc-119(ed3)* III
FR1478	*Si18[hlh-8p::let-418ORF::3xFLAG::let-418 3'UTR + cb-unc-119(+)]* I; *unc-119(ed3)* III (?); *let-418(n3536)* V
FR1485	*Si18[hlh-8p::let-418ORF::3xFLAG::let-418 3'UTR + cb-unc-119(+)]* I; *unc-119(ed3)* III (?); *wIs51[pMF1(scmp::gfp) + pDP#MM016b(unc-119p::unc-119(+)]* V, *let-418(n3536)* V
FR1491	*Si22[rgef-1p::let-418ORF::3xFLAG::let-418 3'UTR + cb-unc-119(+)]* IV; *unc-119(ed3)* III (?); *let-418(n3536)* V
FR1492	*Si18[hlh-8p::let-418ORF::3xFLAG::let-418 3'UTR + cb-unc-119(+)]* I; *unc-119(ed3)* III (?); *let-418(n3536)* V; *ayIs6[pBH47(hlh-8p::gfp) + dpy-20(+)]* X
FR1494	*Si13[elt-2p::let-418ORF::3xFLAG::let-418 3'UTR + cb-unc-119(+)]* I; *daf-2(e1370)* III; *unc-119(ed3)* III (?); *let-418(n3536)* V
FR1511	*daf-2(e1370)* III; *let-418(n3536)* V; *ayIs6[pBH47(hlh-8p::gfp) + dpy-20(+)]* X
FR1513	*Si13[elt-2p::let-418ORF::3xFLAG::let-418 3'UTR + cb-unc-119(+)]* I; *daf-2(e1370)* III; *unc-119(ed3)* III (?); *let-418(n3536)* V; *ayIs6[pBH47(hlh-8p::gfp) + dpy-20(+)]* X
FR1522	*Si18[hlh-8p::let-418ORF::3xFLAG::let-418 3'UTR + cb-unc-119(+)]* I; *daf-2(e1370)* III; *unc-119(ed3)* III (?); *let-418(n3536)* V; *ayIs6[pBH47(hlh-8p::gfp) + dpy-20(+)]* X
FR1523	*Si22[rgef-1p::let-418ORF::3xFLAG::let-418 3'UTR + cb-unc-119(+)]* IV; *unc-119(ed3)* III (?); *let-418(n3536)* V; *ayIs6[pBH47(hlh-8p::gfp) + dpy-20(+)]* X
FR1524	*Si22[rgef-1p::let-418ORF::3xFLAG::let-418 3'UTR + cb-unc-119(+)]* IV; *unc-119(ed3)* III (?); *wIs51[pMF1(scmp::gfp) + pDP#MM016b(unc-119p::unc-119(+)]* V; *let-418(n3536)* V
FR1527	*daf-2(e1370)* III; *ayIs6[pBH47(hlh-8p::gfp) + dpy-20(+)]* X
FR1528	*Si10[pie-1p::let-418ORF::3xFLAG::let-418 3'UTR + cb-unc-119(+)]* I; *daf-2(e1370)* III; *unc-119(ed3)* III (?); *let-418(n3536)* V

Expression constructs

The following plasmids were used in this study:

Plasmid name	Description
p_C33D3.1_93	*elt-2* promoter construct/ pDONRP4-P1R backbone
pCW30	*dpy-7* promoter construct/ pDONRP4-P1R backbone
pCW31	*myo-3* promoter construct/ pDONRP4-P1R backbone
pMS29	*rgef-1* promoter construct/ pDONRP4-P1R backbone
pCG142	*pie-1* promoter construct/ pDONRP4-P1R backbone
pGP6	*let-418* 3'UTR construct/ pDONRP2R-P3 backbone
pSKP-57	*let-418* ORF construct/ pDONR201 backbone
pCFJ150	destination vector containing genomic regions flanking the *ttTi5605* Mos1 insertion
pCFJ210	destination vector containing genomic regions flanking the *ttTi4348* Mos1 insertion
pMS14	*pie-1p::let-418 ORF::3xFLAG::let-418 3'UTR* in pCFJ210
pMS15	*dpy-7p::let-418 ORF::3xFLAG::let-418 3'UTR* in pCFJ210
pMS16	*myo-3p::let-418 ORF::3xFLAG::let-418 3'UTR* in pCFJ210
PMS17	*elt-2p::let-418 ORF::3xFLAG::let-418 3'UTR* in pCFJ210
pMS31	*rgef-1p::let-418 ORF::3xFLAG::let-418 3'UTR* in pCFJ150
pCW51	*hlh-8p::let-418 ORF::3xFLAG::let-418 3'UTR* in pCFJ210

Each expression construct was generated using Multisite Gateway Cloning System (Invitrogen) using plasmids containing a fragment with a tissue-specific promoter (p-C33D3.1_93, pCW30, pCW31, pMS29, pCG142), the coding sequence (containing introns) of *let-418* gene fused to 3x*flag* coding sequence (pSKP-57) and the *let-418* 3'UTR (pGP6), and a MosSCI (Mos1 mediated single copy insertion) compatible destination vector (pCFJ150/pCFJ210). Improved MosSCI technique [1] was used to insert transgenes into a defined site in the *C. elegans* genome. MosSCI transformation was performed based on the protocol described on www.wormbuilder.org/test-page/protocol.

The protocol for antibody-staining of formaldehyde-fixed worms is described on http://www.wormatlas.org/antibodystaining.htm

1. Zeiser, E.; Frokjaer-Jensen, C.; Jorgensen, E.; Ahringer, J. MosSCI and Gateway Compatible Plasmid Toolkit for Constitutive and Inducible Expression of Transgenes in the C. elegans Germline. *PLoS One* **2011**, *6*, e20082, doi:10.1371/journal.pone.0020082 PONE-D-11-03764 [pii].
